# Ustilaginaceae Biocatalyst for Co-Metabolism of CO_2_-Derived Substrates toward Carbon-Neutral Itaconate Production

**DOI:** 10.3390/jof7020098

**Published:** 2021-01-29

**Authors:** Lena Ullmann, An N. T. Phan, Daniel K. P. Kaplan, Lars M. Blank

**Affiliations:** iAMB—Institute of Applied Microbiology, ABBt—Aachen Biology and Biotechnology, RWTH Aachen University, Worringerweg 1, 52074 Aachen, Germany; lena.ullmann@rwth-aachen.de (L.U.); an.phan1@rwth-aachen.de (A.N.T.P.); pramesti.kaplan@rwth-aachen.de (D.K.P.K.)

**Keywords:** itaconate, Ustilaginaceae, *Ustilago maydis*, smut fungi, organic acid, CO_2_, chassis, biodiversity

## Abstract

The family Ustilaginaceae (belonging to the smut fungi) are known for their plant pathogenicity. Despite the fact that these plant diseases cause agricultural yield reduction, smut fungi attracted special attention in the field of industrial biotechnology. Ustilaginaceae show a versatile product spectrum such as organic acids (e.g., itaconate, malate, succinate), polyols (e.g., erythritol, mannitol), and extracellular glycolipids, which are considered value-added chemicals with potential applications in the pharmaceutical, food, and chemical industries. This study focused on itaconate as a platform chemical for the production of resins, plastics, adhesives, and biofuels. During this work, 72 different Ustilaginaceae strains from 36 species were investigated for their ability to (co-) consume the CO_2_-derived substrates acetate and formate, potentially contributing toward a carbon-neutral itaconate production. The fungal growth and product spectrum with special interest in itaconate was characterized. *Ustilago maydis* MB215 and *Ustilago rabenhorstiana* NBRC 8995 were identified as promising candidates for acetate metabolization whereas *Ustilago cynodontis* NBRC 7530 was identified as a potential production host using formate as a co-substrate enhancing the itaconate production. Selected strains with the best itaconate production were characterized in more detail in controlled-batch bioreactor experiments confirming the co-substrate utilization. Thus, a proof-of-principle study was performed resulting in the identification and characterization of three promising Ustilaginaceae biocatalyst candidates for carbon-neutral itaconate production contributing to the biotechnological relevance of Ustilaginaceae.

## 1. Introduction

Itaconic acid is an unsaturated dicarboxylic acid that shows a broad application spectrum due to its two functional groups. It is considered a versatile platform chemical since its derivatives, itaconic acid diamide, 2-methyl-1,4-butanediamine, 2-methyl-1,4-butanediol, 3-methyl-pyrrolidin, 3-methyl-tetrahydrofuran, or unsaturated esters, show potential applications as styrene-butadiene rubbers, synthetic latexes, superabsorbents, unsaturated polyester resins, plastics, coatings, chemical fibers, biofuels, and detergents [1,2,3,4,5,6,7,8,9].

Since the 1950s, industrial biotechnological production of itaconate has been performed by the filamentous fungus *Aspergillus terreus*. This long production and optimization history has enabled titers in a range of 85–100 g L^−1^ and yields near the theoretical maximum at low pH, making *A. terreus*, so far, the best industrial production host for itaconate [10,11,12]. On a laboratory scale, final titers of 160 g·L^−1^ were recently described for *A. terreus* [12]. However, microbial itaconate production using this fungus remains challenging. It shows a production dependent on a certain morphology which is required for its high productivity leading to an increase of the production costs [13,14]. Thus, alternative production hosts are searched. Besides *A. terreus*, other microorganisms have been reported as natural itaconate producers such as yeasts belonging to *Candida* species, smut fungi belonging to the family of Ustilaginaceae such as the pH tolerant *U. cynodontis,* and the yeast-like *Ustilago maydis,* which have recently been engineered to higher efficiency [15,16,17,18]. *U. maydis* itself is a well-studied model organism in biotechnology, plant pathogenicity, and cell biology [19,20,21,22]. Significant improvements on the efficiency of itaconate production in *Ustilago* have been made increasing the yield, titer, and rate of itaconate production in *U. maydis* and related species by metabolic engineering and process development [2,17,23]. For itaconate production from glucose, a maximal theoretical yield of 0.72 g itaconate g glu^−1^, which equals 1 mol itaconate mol glu^−1^ is reported [20]. Thereby, the latest study focused on an integrated process design that resulted in a maximum itaconate titer of 220 g L^−1^, with a total acid titer of 248 g L^−1^, which displayed a significant improvement compared to the best-published itaconate titers reached with *U. maydis* and with *A. terreus* [2].

Nevertheless, progress in biotechnology and microbial itaconic acid production is required to further replace petroleum-based products. In 2011, the market size of itaconic acid was relatively small, with 41,400 tons and a market value of USD 74.5 million [24]. This was caused by the relatively high price of approximately two dollars per kg and the availability of cheaper petro-based alternatives such as acrylic acid. To be competitive against petro-based products and access further markets, costs need to reduce to around USD 0.5 per kg [25]. Assuming a price decrease, itaconic acid could replace acrylic acid in the production of poly(methyl methacrylate), the production of which is petroleum-based with a market worth USD 11 billion [1,5,14].

The competition against petro-based products displays a first step toward the vision of a circular economy. Furthermore, utilization of every production side stream to minimize waste and ultimately CO_2_ formation is required [26]. To achieve economic production and to compete with fossil fuels, it is necessary to use low-cost feedstocks. Current biotechnological processes for itaconic acid production with *A. terreus* and *U. maydis* are based on carbohydrates, such as molasses, xylose, arabinose, and glucose [10,27]. Furthermore, glycerol from biodiesel is an alternative feedstock for the itaconate production process using *U. vetiveriae* [18,28]. To improve the sustainability of the itaconate production process, a new approach with potential CO_2_-derived C1 and C2 compounds (formate and acetate) as co-substrates is needed to reduce the carbon footprint, potentially toward the overall goal of a carbon-neutral itaconic acid production process.

Formate recently gained interest as a carbon source that can be readily synthesized from CO_2_ via (electro-)chemical catalysis [29]. In contrast, acetate can be synthesized by acetogens from CO_2_ and H_2_, in combination with glucose at rates of about 30 mmol g_CDW_^−1^ h^−1^ [30,31]. Furthermore, research on energy storage producing chemically stable and valuable products using CO_2_ feedstocks was carried out focusing on microbial electrosynthesis (MES). Thereby, acetate was produced at 61% Coulombic efficiency and fully recovered as an acidified stream containing up to 13.5 g L^−1^ (225 mM) acetic acid [32].

The acetate assimilation mechanisms in yeasts were already identified [33]. Acetate can enter the cell through passive diffusion, although several transporters are implicated in the uptake of acetate into the cell [34,35]. It serves as a substrate for the enzyme acetyl-CoA synthase (ACS), which converts acetate to acetyl-CoA in the cytosol [33]. The acetate assimilation for *U. maydis* has not been well researched yet, but the assimilation in yeasts may give a similar overview since acetyl-CoA synthase (ACS) exists in *U. maydis* as well [36]. According to Kretschmer et al., different acetate uptake mechanisms occur depending on extracellular pH [37]. This study observed that activation of long-chain fatty acids and acetate as a growth-dependent carbon source depends on peroxisomal activation to short acyl-CoAs. This includes acetyl-CoA and its shuttling to the mitochondria via carnitine [37]. Furthermore, growth on acetate as a sole carbon source was shown [37]. Demonstrating the connection between acetate and β-oxidation in *U. maydis,* it was observed that certain deletion mutants, e.g., defective in *had1* gene, encoding the mitochondrial β-oxidation enzyme hydroxyacyl coenzyme A dehydrogenase, were unable to grow on acetate [37].

**Figure 1:**
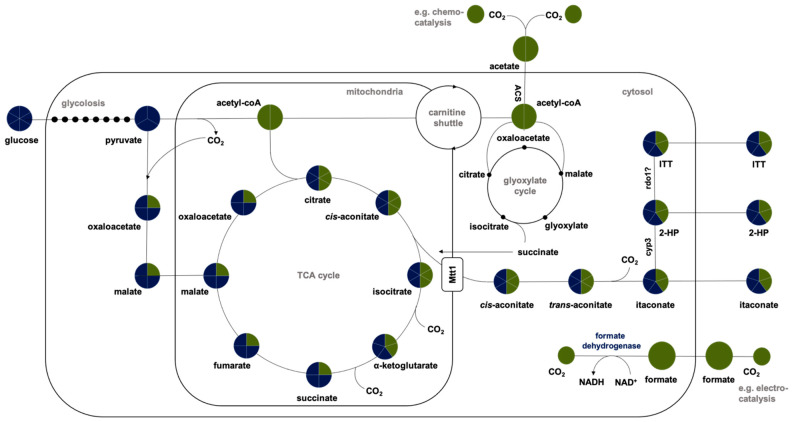
Itaconate biosynthesis pathway in *Ustilago maydis* with a proposed acetate and formate assimilation. Pyruvate is generated from glucose through glycolysis taking place in the cytoplasm. It enters the mitochondria, where it is converted to acetyl-CoA and forms citrate together with oxaloacetate during the TCA cycle. Citrate is dehydrated to *cis*-aconitate which is transported from the mitochondria into the cytosol via the mitochondrial tricarboxylate transporter Mtt1. In the cytosol, *cis*-aconitate is converted into itaconate via the intermediate *trans*-aconitate. Itaconate can be further converted to 2-hydroxyparaconate (2-HP) by Cyp3. 2-hydroxyparaconate might be converted to itatartarate (ITT) by Rdo1. Secretion of itaconate and possibly 2-hydroxyparaconate and itartarate into the medium is mediated by the major facilitator Itp1. Modified from [20,38]. Proposed acetate assimilation modified from [33,37]. Acetate enters the cell via passive diffusion and/or via putative acetate transporters [34,35,37]. It serves as a substrate for the enzyme acetyl-CoA synthase (ACS) [36], which converts acetate to acetyl-CoA in the cytosol [33]. Growth on acetate depends on peroxisomal activation to short acyl-CoAs including acetyl-CoA and its shuttling to the mitochondria via carnitine [37]. Proposed formate assimilation via formate dehydrogenases is known for methylotrophic microorganisms [39]. These enzymes are also present in *U. maydis* [40]. Indicated circle segments represent the number of carbon atoms per molecule. Blue circles indicate carbon derived from conventional glucose whereas green color indicates carbon possibly derived from CO_2_.

A proposed pathway for acetate and formate assimilation in *U. maydis*-incorporating itaconic acid production is shown in Figure 1. Thereby, the itaconate biosynthesis pathway in *U. maydis* and the corresponding genes are identified and well-characterized [20,38]. Pyruvate is generated from glucose through glycolysis taking place in the cytoplasm. It enters the mitochondria, where it is converted to acetyl-CoA and forms citrate together with oxaloacetate in the TCA cycle. Citrate is dehydrated to *cis*-aconitate which is transported from the mitochondria into the cytosol via the mitochondrial tricarboxylate transporter Mtt1. In the cytosol, *cis*-aconitate is converted into itaconate via the intermediate *trans*-aconitate. Itaconate can be further converted to 2-hydroxyparaconate by Cyp3. Secretion of itaconate and possibly 2-hydroxyparaconate and itatartarate (ITT) into the medium is mediated by the major facilitator Itp1 [20,38]. During the conventional itaconate production process using glucose, the theoretical stoichiometry is glucose equals itaconate plus CO_2_. In contrast, the theoretical stoichiometry of the acetate co-feeding process is glucose plus four CO_2_ equals two molecules of itaconate.

Formate degradation via formate dehydrogenases (FDHs) is present in all methylotrophic microorganisms, which can perform oxidations of formate to CO_2_ as one of the main sources of energy in the form of NADH. NAD^+^-dependent FDHs were found in all yeasts of the genera *Candida, Pichia*, and *Hansenula* using methanol and were isolated and characterized from different strains [39]. Even though it is not a methylotrophic organism, these enzymes are also present in *U. maydis* [40]. So far, formate degradation via formate dehydrogenases (FDHs) is not characterized in such detail for *U. maydis.* Nevertheless, we proposed that the FDH activity in the cytosol is similar to the mechanism in *Saccharomyces cerevisiae* described by Overkamp et al. [41]. Besides native metabolic routes, synthetic pathways display a valuable tool such as Gonzalez de la Crus et al. previously identified the synthetic reductive glycine pathway as the most efficient route for aerobic growth on formate [42].

The goal of this study was to develop Ustilaginaceae biocatalysts for co-metabolism of CO_2_-derived substrates toward carbon-neutral itaconate production. Acetate and formate as carbon sources recently gained interest [43,44] but their utilization remains challenging. Acetate shows toxicity and inhibitory effects on many microorganisms whereas even co-utilization with glucose challenges the underlying regulatory networks of metabolism favoring glucose utilization [45,46,47,48]. Here, to exploit nature’s biodiversity, 72 different Ustilaginaceae of 36 species were tested for acetate and formate use. Growth on substrate mixtures and product spectrum with special interest in itaconate was characterized. Thereby, *U. maydis* and *U. rabenhorstiana* were identified as promising candidates for acetate metabolization whereas *U. cynodontis* was identified as a potential production host enhancing its itaconate production by the use of formate as a co-substrate. Selected strains with the highest itaconate production were further characterized in controlled-batch cultivation experiments confirming the trends observed in small scale cultivations. Furthermore, extracellular metabolites were identified enabling future metabolic engineering strategies. Thus, a proof-of-principle study was performed resulting in the identification and characterization of three promising Ustilaginaceae biocatalyst candidates for carbon-neutral itaconate production contributing to the biotechnological relevance of Ustilaginaceae.

## 2. Materials and Methods

### 2.1. Culture Conditions

Growth and production experiments were performed using modified Tabuchi medium according to Geiser et al. [20] containing 0.2 g L^−1^ MgSO_4_·7H_2_O, 0.01 g L^−1^ FeSO_4_·7H_2_O, 0.5 g L^−1^ KH_2_PO_4_, 1 mL L^−1^ vitamin solution, 1 mL L^−1^ trace element solution, and as buffer 19.5 g L^−1^ 2-(N-morpholino) ethanesulfonic acid (MES). Different carbon sources such as glucose, sodium acetate, and sodium formate were used as well as the c-source concentrations varied in different experiments. NH_4_Cl was added in indicated concentrations. The vitamin solution contained (per liter) 0.05 g D-biotin, 1 g D-calcium pantothenate, 1 g nicotinic acid, 25 g myo-inositol, 1 g thiamine hydrochloride, 1 g pyridoxol hydrochloride, and 0.2 g para-aminobenzoic acid. The trace element solution contained (per liter) 1.5 g EDTA, 0.45 g ZnSO_4_·7H_2_O, 0.10 g MnCl_2_·4H_2_O, 0.03 g CoCl_2_·6H_2_O, 0.03 g CuSO_4_·5H_2_O, 0.04 g Na_2_MoO_4_·2H_2_O, 0.45 g CaCl_2_·2H_2_O, 0.3 g FeSO_4_·7H_2_O, 0.10 g H_3_BO_3_, and 0.01 g KI. Cultivation experiments were performed at 30 °C.

Screening for biomass formation was performed using the Growth Profiler GP960 (EnzyScreen, Heemstede, the Netherlands) [49]. Strains were cultivated in polystyrene grey square 24-deep-well microplates (CR1424d) with a transparent bottom and a filling volume of 1.5 mL (225 rpm, d = 50 mm). Acetate and formate were used as co-substrates in concentrations of 2.5, 5.0, and 10.0 g L^−1^ in combination with 20.0 g L^−1^ glucose. Growth on 20 g L^−1^ glucose, 5 g L^−1^ acetate, or 5 g L^−1^ formate was tested separately as a respective reference. Growth experiments were performed using 4 g L^−1^ NH_4_Cl. For the screening experiments, cells with final OD_600nm_ of 0.5 were used from an overnight culture in modified Tabuchi medium (MTM) containing 20 g L^−1^ glucose as an inoculum [20].

Cultivations in connection with itaconic acid production were performed in System Duetz^®^ (24 deep-well microtiter plates, EnzyScreen, Heemstede, the Netherlands) with a filling volume of 1.5 mL (300 rpm, 80% humidity, d = 50 mm, Infors HT Multitron Pro shaker, Bottmingen, Switzerland) [49]. Cultures were inoculated in parallel into multiple microtiter plates to a final OD_600nm_ of 0.5 with cells from an overnight culture in MTM medium containing 50 g L^−1^ glucose. For each sample point, a complete plate was taken as a sacrificial sample to ensure continuous oxygenation. Therefore, samples for analytical methods (see Section 2.3) were taken at 6–8 timepoints distributed throughout the experiment approximately every 24 h. Experiments were terminated after 120–170 h when a decreasing itaconate production was observed. Preliminary production experiments were performed with 20 g L^−1^ glucose and 2.5 g L^−1^ co-substrate. Afterward, concentrations were increased to 50.0 g L^−1^ glucose and 6.25 g L^−1^ co-substrate. Cultivation experiments were performed using 0.8 g L^−1^ NH_4_Cl.

Controlled-batch cultivations were performed in a BioFlo^®^ 120 bioreactor with a total volume of 1.3 L and a working volume of 0.5 L in combination with DASware Control Software 5.3.1 (Eppendorf, Hamburg, Germany). Cultivations were performed in batch medium containing 50 L^−1^ glucose, in combination with 6.25 g L^−1^ co-substrate, 0.8 g L^−1^ NH_4_Cl, 0.2 g L^−1^ MgSO_4_·7H_2_O, 0.01 g L^−1^ FeSO_4_·7H_2_O, 0.5 g L^−1^ KH_2_PO_4_, 1 g L^−1^ yeast extract, 1 mL L^−1^ vitamin solution, 1 mL L^−1^ trace element solution, and 19.5 g L^−1^ MES as buffer. During cultivation, pH was monitored via online pH probes (phferm, Hamilton Company, Bonaduz, Switzerland) and maintained at pH 6.5 by automatic addition of 10 M NaOH and 1 M HCl. Dissolved oxygen tension (DOT) was kept constant at approximately 80% saturation by automatic adjustment of the stirring rate (800–1200 rpm). The bioreactor was aerated with an aeration rate of 1 L min^−1^ (2 vvm), while evaporation was limited by sparging the air through a water bottle. The temperature was set at 30 °C. The bioreactor was inoculated to a final OD_600_ of 0.5 with cells from an overnight culture in 50 mL MTM containing 50 g L^−1^ glucose and 6.25 g L^−1^ of respective co-substrate. A pulsed fed-batch bioreactor experiment was performed in the previously described batch medium containing 200 g L^−1^ glucose and in total 25 g L^−1^ acetate. Thereby, acetate was added three times during the process at 15, 63, and 91 h. The bioreactor was inoculated to a final OD_600_ of 0.5 with cells from an overnight culture in 50 mL MTM containing 50 g L^−1^ glucose. Samples for analytical methods (see Section 2.3) were taken at the latest every 24 h during all bioreactor experiments whereas sampling frequency was shorter during the beginning of the cultivation.

### 2.2. Strain Selection

72 strains of the Ustilaginaceae family were screened in this study (Table S1, Supplementary Materials). Numbers behind the species name indicate the strain number. The biodiversity screening procedure was performed via a three-step ranking process. Firstly, the tested strains were sorted ordinally according to their maximum OD_600_ in the respective co-substrate category (acetate, formate) and concentrations (2.5, 5.0, and 10.0 g L^−1^). Secondly, strains with a higher growth compared to the respective glucose reference were selected resulting in ranking the best ten Ustilaginaceae strains for each co-substrate. Despite the ability to cope with the different co-substrates and concentrations, the itaconic acid production based on literature research [15,18,19] was taken into account during the third-ranking step. Thereby all co-substrate concentration categories were considered, resulting in the best five strains for each co-substrate.

During production experiments, the ten best-performing strains selected from biodiversity screening were tested. Additionally, *U. maydis* #2229 was used in the experimental set-up as a reference strain, as it displays the wildtype strain of multiple metabolically engineered itaconate chassis strains [2,23].

Controlled-batch cultivation experiments were conducted using the three most promising Ustilaginaceae candidates, *U. maydis* #2229, *U. rabenhorstiana* #2708, and *U. cynodontis* #2705 obtained from small-scale production experiments.

### 2.3. Analytical Methods

Cell growth was determined by measuring the optical density at 600 nm (OD_600_) with an Ultrospec 10 Cell Density Meter (Amersham Biosciences, Buckinghamshire, UK). The majority of cells showed single-cell yeast-like morphology. Nevertheless, during the experimental procedure, strains showing prominent filamentous growth were excluded as strains were sought that grow the best under certain conditions.

Carbon sources and metabolites such as glucose, acetate, formate, itaconate, malate, succinate, erythritol, and (S)-2-hydroxyparaconate in the supernatant were analyzed via high-performance liquid chromatography (HPLC). Therefore 1 mL culture broth was centrifuged at maximum speed (Heraeus Megafuge 16 R, TX-400 rotor, Thermo Scientific, Waltham, MA, USA). The supernatant was filtered with Rotilabo^®^ syringe filters (CA, 0.20 µm) and afterward diluted in a range of 1:5–1:50 with 5 mM H_2_SO_4_. Supernatants were analyzed in a DIONEX UltiMate 3000 HPLC System (Thermo Scientific, Waltham, MA, USA) with a Metab-AAC column (300 × 7.8 mm column, ISERA, Düren, Germany). Elution was performed with 5 mM H_2_SO_4_ at a flow rate of 0.6 mL, min^−1^ and a temperature of 40 °C. For detection, a SHODEX RI-101 detector (Showa Denko Europe GmbH, München, Germany) and a DIONEX UltiMate 3000 Variable Wavelength Detector set to 210 nm were used.

The identification of other extracellular metabolites than the abovementioned was performed on a Nexera UHPLC system (Shimadzu Corporation, Kyōto, Japan) with 0.2% formic acid as eluent. After the samples were separated on an Isera Metab-AAC 300 × 7.8 mm column (ISERA, Düren, Germany), the flow was divided into 2 directions with the split ratio of 1 to 10. The major part of the samples was measured with a RID-20A Refractive Index detector and an SPD-40 UV detector at 210 nm (Shimadzu Corporation, Kyōto, Japan). The rest were analyzed with a triple quadrupole mass spectrometry 8060 (Shimadzu Corporation, Kyōto, Japan). The retention time from all detectors and the MS/MS mass spectrums of samples were compared directly to authentic standards. As the standard was not available for itatartarate, the structure prediction was performed with the software CFM-ID 3.0 [50].

All values are the arithmetic mean of at least two biological replicates. Error bars indicate the deviation from the mean for *n* = 2, if *n* > 2 error bars indicate the standard error of the mean. Statistical significance was evaluated by t-test (two-tailed distribution, heteroscedastic, *p* ≤ 0.05). Hierarchical cluster analysis (HCA) was performed using the MultiExperiment Viewer (MeV) [51]. Due to the high number of 1296 growth curves obtained during this study, a MatLab function modified from [52] was used for standardized maximum growth rate calculation. Itaconate product yields were calculated as stated in Y_P/S_ [g_ITA_/g_c-source_] and Y_P/S_ [C-moL_ITA_/C-moL_c-source_] in order to equalize c-source concentration effects on itaconic acid product yields according to Geiser et al. [20].

## 3. Results and Discussion

### 3.1. Biodiversity Screening for Growth on Acetate and Formate in Combination with Glucose

**Figure 2:**
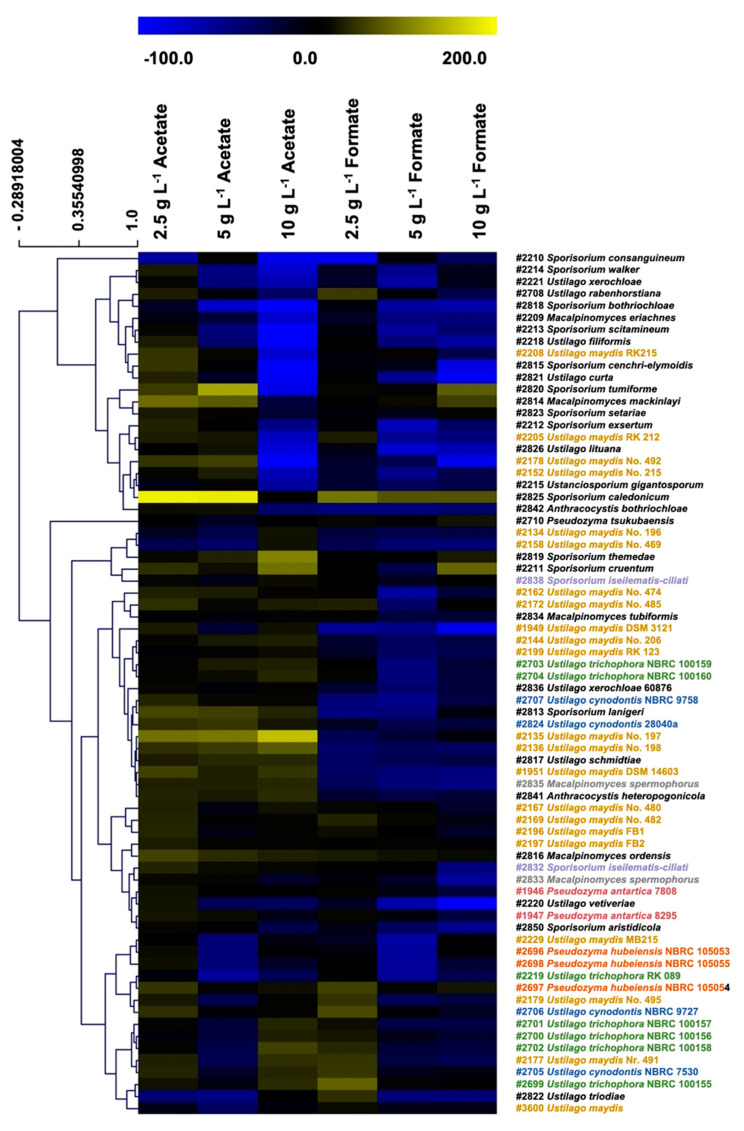
Overview of biodiversity screening results. Hierarchical cluster analysis (HCA) heatmap showing whole set growth screening results obtained during 24-deep-well plate cultivation in MTM medium with 4 g L^−1^ NH_4_Cl using the Growth Profiler system by EnzyScreen. Strains were cultivated for growth on both co-substrates under various conditions of 2.5, 5.0, and 10.0 g L^−1^ in combination with 20.0 g L^−1^ glucose. Maximum optical density (OD_600_) was normalized to the growth of the respective glucose reference and visualized via color scales in the HCA heatmap indicating relative growth [%] Blue color indicates a lower growth, black a comparable growth behavior, and yellow a higher growth compared to the respective glucose reference. Strains belonging to one species were colored accordingly in the displayed rows. Experiments were performed with two biological duplicates. Raw data are provided in Supplementary Materials (Tables S2 and S3).

For the identification of promising biocatalysts contributing to a CO_2_-neutral synthesis of itaconic acid, 72 different Ustilaginaceae strains of 36 species in total were cultivated and screened for growth on acetate and formate as potential co-substrates derived from CO_2_. Strains were cultivated on different concentrations of both co-substrates (2.5, 5.0, and 10.0 g L^−1^) in combination with 20.0 g L^−1^ glucose. Promising candidates were considered as those growing under a desirably high concentration of either of these co-substrates while achieving a higher maximal biomass concentration compared to their growth on glucose only (Figure 2).

Via HCA, distinct co-substrate effects on the different Ustilaginaceae strains were revealed. Higher co-substrate concentrations entailed a decrease of the maximum OD_600_ and the growth rates for most of the tested strains (Tables S2 and S3), whereas formate, in general, showed a stronger inhibitory effect on microbial growth compared to acetate. While the addition of 2.5 g L^−1^ acetate led to reduced biomass concentrations in one-third of the tested Ustilaginaceae, the addition of the same concentration of formate reduced the maximal OD_600_ in 75% of the strains. The highest co-substrate concentration of 10 g L^−1^ led to a decrease of 69% of all strains with acetate and 97% using formate as a co-substrate. These results confirm previous studies where acetate showed toxicity and inhibitory effects on many microorganisms [45,46,52,53]. One factor impacting cell growth of the tested Ustilaginaceae might be the pH shift during cultivation starting from pH 6.5 shifting up to a maximum pH of 8.6 when acetate or formate are metabolized. pH values were determined at the end of the cultivation experiments, and raw values are provided in the Supplementary Materials section (Table S4). Calculating the average pH values of all 72 strains for each tested condition resulted in 5.7 ± 0.2 (glucose reference), 6.3 ± 0.3 (2.5 g L^−1^ acetate), 7.0 ± 0.4 (5 g L^−1^ acetate), 8.3 ± 0.7 (10 g L^−1^ acetate), 6.5 ± 0.2 (2.5 g L^−1^ formate), 7.4 ± 0.7 (5 g L^−1^ formate), and 7.9 ± 0.8 (10 g L^−1^ formate). Usually, microorganisms prefer a limited and specific pH range. Furthermore, smut fungi are known to grow filamentous under non-optimal growth conditions [17].

Nevertheless, acetate and formate are known to have the following effects on microorganisms. According to Kretschmer et al., acetate provokes mitochondrial stress in *U. maydis*. Higher concentrations of acetate not only cause acidification of the cytosol, leading to impaired enzyme activity, initiation of programmed cell death, and increased levels of reactive oxygen species (ROS), but also reduce the expression of ROS detoxification mechanisms, boosting oxidative stress further [37]. The insights of Lastauskienė et al. are comparable in terms of the effects of formate on *Candida* species. Formate inhibits the cytochrome-c-oxidase, which is responsible for maintaining a proton gradient by oxidizing cytochrome-c and by reducing oxygen to water. Protons resulting from the formate catalyzation are transferred into the mitochondrial intermembrane space ensuring ATP synthesis through ATP-synthase [54]. The described issues could be tackled with specific feeding strategies such as fed-batch or with a pH-control during bioreactor fermentations. Thus, the decreasing trend in the maximum optical density (OD_600_) and growth rate could be explained by the previously described inhibiting effects of acetate and formate, especially with higher co-substrate concentrations.

**Figure 3:**
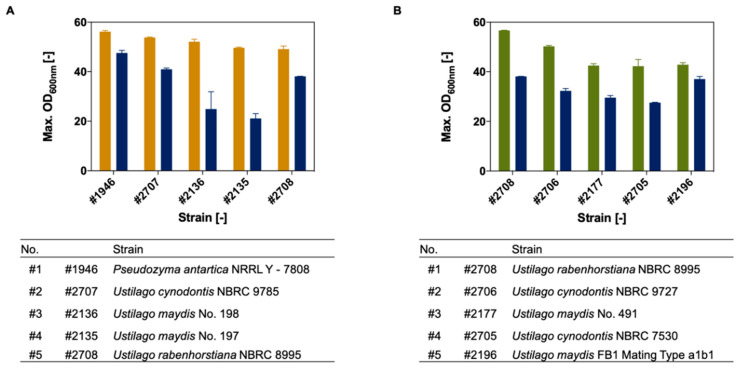
Maximum growth of best Ustilaginaceae candidates obtained from biodiversity screening. (**A**) The top 5 Ustilaginaceae candidates using acetate as a co-substrate (orange). #2707, #1946, and #2708 obtained the highest growth using 2.5 g L^−1^ acetate whereas #2135 and #2136 obtained the best results using 10 g L^−1^ acetate. (**B**) The top 5 Ustilaginaceae strains using 2.5 g L^−1^ formate as a co-substrate (green). Growth Profiler 24-deep-well plate cultivation was performed in MTM medium with 4 g L^−1^ NH_4_Cl. Respective glucose references (20 g L^−1^) are shown in blue. Error bars indicate the deviation from the mean for *n* = 2.

Further observations during HCA analysis could be made concerning the biodiversity among the tested smut fungi of 36 different species in total. Twenty-two *U. maydis* strains were tested for growth on acetate and formate whereas no clear trend was observed for all strains as they are distributed all over the clusters (Figure 2). This finding is encouraged by a previous study from Geiser et al. that showed a high variation in the itaconate production of 52 different *U. maydis* strains [15]. In contrast, certain strains showed a similar trend, and therefore they were clustered close to each other on the HCA plot. *U. maydis* #1951, #2135, and #2136 showed a higher growth on acetate but, in contrast, did not grow well on formate compared to their respective glucose reference. Four other *U. maydis* strains, #2167, #2169, #2196, and #2197, were grouped close together. Those strains showed better performance on formate compared to the previously discussed group. In addition, seven different *U. trichophora* strains were tested during this study which showed a less broad distribution compared to the *U. maydis* strains. Except for the two strains *U. trichophora* #2703 and #2704, the remaining five strains were clustered relatively close to each other on the plot. Furthermore, the tested *U. cynodontis* strains #2705 and #2706 were clustered relatively close to each other indicating a similar co-substrate utilization pattern. Both strains were later picked as candidates for best formate utilization (Figure 3). In total, three different *Pseudozyma hubeiensis* strains #2696, #2696, and #2698 were tested, showing a close clustering in the plot. Nevertheless, due to the broad biodiversity and different tested co-substrate conditions, the interpretation of clearly differentiated clusters and their trends remains challenging. Thus, no distinct correlation between the strains’ abilities to grow utilizing different co-substrates and their evolutionary relationships could be identified.

To identify suitable biocatalysts for the co-utilization of acetate and formate, a three-step ranking process was performed. Thereby, Figure 3 displays the final Ustilaginaceae candidates which can utilize acetate or formate as co-substrate. Within the best acetate utilizers, the strains *U. maydis* #2135 and #2136 showed the highest growth increase upon the addition of 10 g L^−1^ acetate. In contrast, the best candidates using formate showed the best results using the lowest formate concentration of 2.5 g L^−1^. Growth results of the best ten strains and their biomass yields are displayed in Table S5 for acetate and in Table S6 for formate.

The most promising Ustilaginaceae candidates determined using acetate as a co-substrate are stated as follows according to their max. OD_600_: *Pseudozyma antartica* #1946 (max. OD_600_ 56 ± 2/growth increase compared to glucose + 18%), *U. cynodontis* #2707 (54 ± 1/+31%), *U. maydis* #2136 (51 ± 2/+118%), *Ustilago maydis* #2135 (50 ± 0/+136%), and *U. rabenhorstiana* #2708 (48 ± 2/+29%). Formate ranking determined the following best five strains sorted by OD_600_: *U. rabenhorstiana* #2708 (57 ± 0/+49%), *U. cynodontis* #2706 (51 ± 0.5/+56%), *U. maydis* #2177 (42 ± 1/+44%), *Ustilago cynodontis* #2705 (40 ± 4/+53%), and *U. maydis* #2196 (42 ± 1/+16%). *Ustilago rabenhorstiana* #2708 attracted special attention, as it is ranked as one of the best five strains for each co-substrate.

### 3.2. Screening for Best Itaconate Producers Using Acetate and Formate as Co-Substrates

Given biodiversity screening results, the production of organic acids with special interest in itaconic acid was further investigated. Production of itaconate, and many other secondary metabolites, is induced by nitrogen limitation in *U. maydis* [19,55,56].

When using experimental conditions similar to the biodiversity screening (20 g L^−1^ glucose, 2.5 g L^−1^ acetate), titers of up to 1.6 g L^−1^ itaconate were obtained which is displayed in Figure S1. By increasing the carbon source concentrations, maximum of 7.6 g L^−1^ itaconate was obtained. The increased carbon source concentrations resulted in up to 2-fold higher product yields. Thereby, the maximum obtained yield among the strains was 0.07 ± 0.0 Y_P/S_ [g_ITA_/g_c-source_] corresponding to the low carbon source concentration vs. 0.15 ± 0.0 in the presence of the higher carbon source concentration. Nevertheless, the same trends were observed regarding the co-substrate utilization and itaconate production, i.e., strains that perform well at low carbon source concentrations also perform well at high carbon source concentrations. Thus, testing for best itaconate producers was continued with the high carbon source concentration.

**Figure 4:**
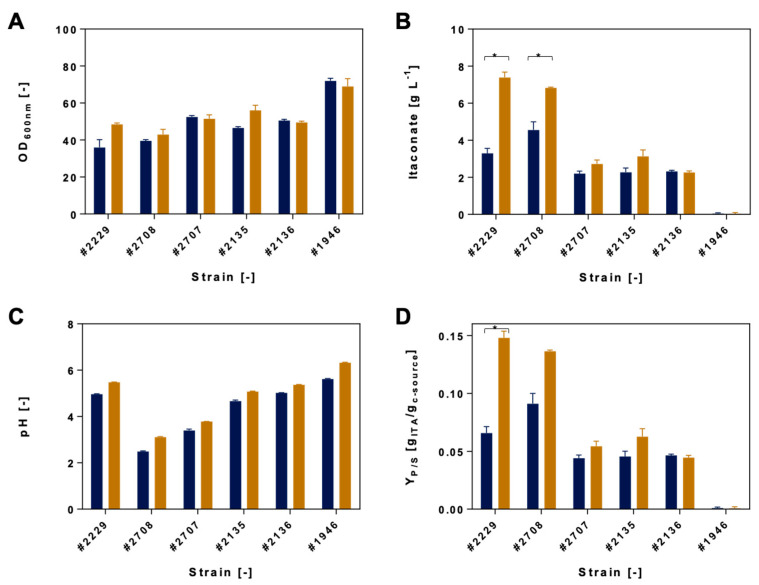
Itaconic acid production of selected Ustilaginaceae strains. (**A**) Maximum optical density (OD_600_ [−]), (**B**) maximum itaconic acid production [g L^−1^], (**C**) minimum pH [−], and (**D**) Y_P/S_ [g_ITA_/g_c-source_] during System Duetz^®^ 24-deep-well plate cultivation experiments with 1.5 mL MTM medium and 0.8 g L^−1^ NH_4_Cl. Ustilaginaceae candidates using acetate as a co-substrate are shown in orange (6.25 g L^−1^). Respective glucose references (50 g L^−1^) are shown in blue. Error bars indicate the deviation from the mean for *n* = 2. Statistically significant differences in itaconic acid production (*p* ≤ 0.05) are indicated as *. Details of statistical analyses are displayed in Tables S11 and S12.

**Figure 5:**
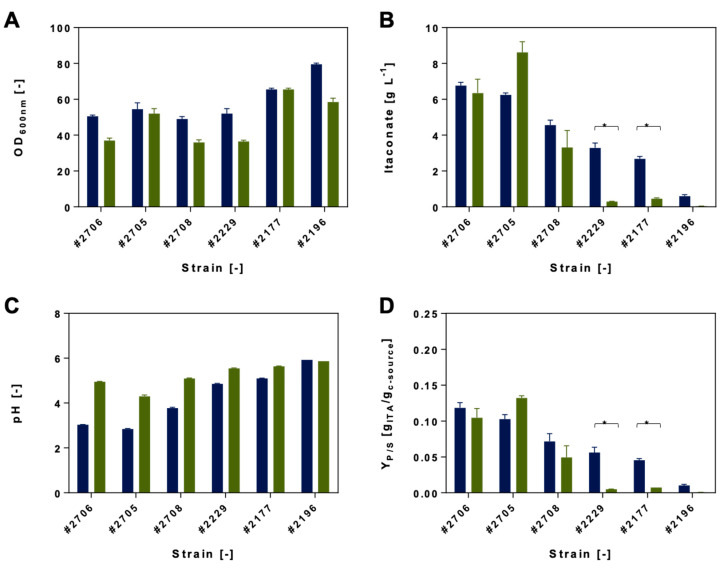
Itaconic acid production of selected Ustilaginaceae strains. (**A**) Max. optical density (OD_600_ [−]), (**B**) maximum itaconic acid production [g L^−1^], (**C**) minimum pH [−], and (**D**) Y_P/S_ [g_ITA_/g_c-source_] during System Duetz^®^ 24-deep-well plate cultivation experiments with 1.5 mL MTM medium and 0.8 g L^−1^ NH_4_Cl. Ustilaginaceae candidates using formate as a co-substrate are shown in green (6.25 g L^−1^). Respective glucose references (50 g L^−1^) are shown in blue. Error bars indicate the deviation from the mean for *n* = 2. Statistically significant differences in itaconic acid production (*p* ≤ 0.05) are indicated as *. Details of statistical analyses are displayed in Tables S11 and S12.

An overview of the itaconic acid production screening results of the most promising Ustilaginaceae strains is displayed in Figure 4 and Figure 5. The utilization of acetate and formate will be discussed separately. Detailed production parameters are listed in Tables S7–S10.

#### 3.2.1. Itaconate Production Using Acetate

Compared to biodiversity screening results, itaconic acid production experiments resulted in a different outcome. *U. maydis* #1946, which obtained the highest growth using acetate as a co-substrate, did not produce a significant amount of itaconate. Furthermore, 0.1 ± 0.0 g L^−1^ itaconate was produced using 50 g L^−1^ glucose, while cultivation with acetate resulted in a decrease toward 0.01 ± 0.0 g L^−1^. In contrast, max. OD_600_ increased from 60 ± 5 to 69 ± 7. Thus, this strain might use the co-substrate for biomass formation rather than for itaconate production.

The two strains *U. maydis* #2229 and *U. rabenhorstiana* #2708 performed best in System Duetz cultivation, reaching itaconate titers of 7.4 ± 0.3 g L^−1^ and 6.8 ± 0.1 g L^−1^, respectively, which corresponds to a 2.2-fold and 1.6-fold increase of the production.. *U. maydis* #2229 showed a 2.3-fold increase based on Y_P/S_ [g_ITA_/g_c-source_] and a 2.1-fold increase based on Y_P/S_ [C-moL_ITA_/C-moL_c-source_]. Total itaconate production of *U. cynodontis* #2707, *U. maydis* #2135, and *U maydis* #2136 was observed in a range between 2 and 3 g L^−1^ itaconate in the presence of acetate: 2.9 ± 0.0 g L^−1^, 3.3 ± 0.4 g L^−1^, and 2.3 ± 0.1 g L^−1^. The itaconate titer reached by *U. maydis* #2136 decreased compared to the cultivation on glucose only (2.8 ± 0.6 g L^−1^). Nevertheless, two promising strains were identified which reached higher product titers using acetate as a co-substrate—*U. maydis* #2229 and *U. rabenhorstiana* #2708—which were further characterized during controlled-batch fermentations.

#### 3.2.2. Itaconate Production Using Formate

Comparing formate conditions of the biodiversity screening and itaconate production results, differences can be drawn among the tested strains. Except for *U. cynodontis* #2705, the tested strains reached lower itaconate titers when formate was present during cultivation. Formate was not only not used for itaconate production, but it interfered with the production. The reference strain *U. maydis* #2229 exhibited a reduced itaconate titer of 0.3 ± 0.0 g L^−1^ (7.4-fold decrease). *U. maydis* #2177 and #2196 did show a drastic decrease in itaconate production as well. A shifting pH effect toward alkaline values during formate cultivations was observed, probably contributing to reduced itaconate titers (Table S10).

In general, *U. cynodontis* was identified as one of the best itaconate-producing species by Hosseinpour Tehrani et al. [17]. Given the results, *U. cynodontis* #2705 is considered the most promising strain metabolizing formate for itaconate production. In contrast to acetate which can be directly used as a carbon and energy source, formate co-consumption delivers extra electrons to the fungal metabolism. Using formate, an itaconate production titer of 8.6 ± 0.6 g L^−1^ was observed (Table S8). Furthermore, filamentous growth was observed. Nevertheless, this can be avoided by metabolic engineering and deletion of the genes *ras2, fuz7,* or *ubc3* of the MAPK signal cascade shown by Hosseinpour Tehrani et al. for *U. cynodontis* [22].

During the performed screening for the best itaconate producers using acetate or formate as co-substrates, the following strains were considered as promising candidates and were used for subsequent experiments: *U. maydis* #2229 and *U. rabenhorstiana* #2708 for acetate co-metabolism and *U. cynodontis* #2705 for formate co-metabolism.

### 3.3. Controlled-Batch Fermentation of the Best Itaconate Producers

**Figure 6:**
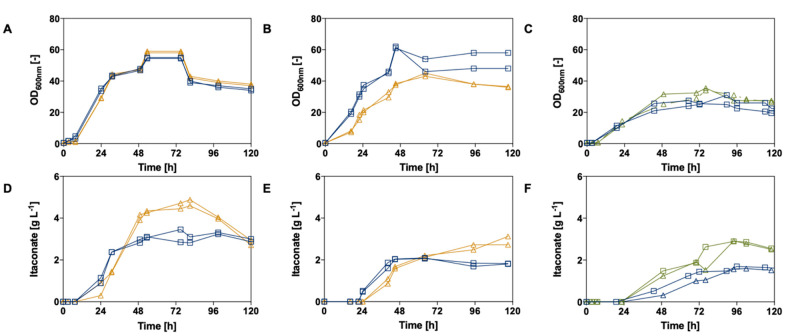
Controlled-batch fermentations of selected Ustilaginaceae candidates. OD_600_ of (**A**) *U. maydis* #2229, (**B**) *U. rabenhorstiana* #2708, and (**C**) *U. cynodontis* #2705. Itaconate production is shown for (**D**) *U. maydis* #2229, (**E**) *U. rabenhorstiana* #2708, and (**F**) *U. cynodontis* #2705 during fermentation in a bioreactor containing MTM medium (0.8 g L^−1^ NH_4_Cl, 30 °C, 80% DOT, at pH 6.5). Ustilaginaceae candidates using acetate as a co-substrate are shown in orange, and formate co-substrate cultivations are shown in green (6.25 g L^−1^). Respective glucose references (50 g L^−1^) are shown in blue.

To further investigate and confirm itaconic acid production of the three most promising Ustilaginaceae candidates, *U. maydis* #2229, *U. rabenhorstiana* #2708, and *U. cynodontis* #2705 were cultivated in controlled-batch fermentation (Figure 6, Table 1). Thereby, cultivation conditions remained similar to small-scale production experiments.

By comparing the controlled-batch cultivation differences in growth, phases of the tested organisms appear. Substrate consumptions are displayed in Figure S2. In general, additional acetate and formate were consumed simultaneously with glucose, and no diauxic growth or metabolic adaption was observed. Nevertheless, the glucose consumption was prolonged with the addition of a co-substrate. *U. maydis* #2229 consumed glucose within 31 h compared to 53 h in the presence of acetate. *U. rabenhorstiana* #2708 depleted glucose within 48 h compared to doubling consumption times of 95 h under co-substrate conditions. *U. cynodontis* #2705 showed a longer growth phase in comparison to the acetate cultivations of 92 h compared to 100 h using formate as a co-substrate. In contrast, 6.25 g L^−1^ acetate was consumed within 24 h by *U. maydis* and 40 h by *U. rabenhorstiana*. Formate depletion was observed after 118 h for *U. cynodontis.*

During bioreactor experiments, the strains produced metabolites such as itaconate, malate, erythritol, and succinate under nitrogen limitation. Total itaconic acid concentrations are displayed in Table 1 and were observed as the following: 4.7 ± 0.2 g L^−1^ for *U. maydis* #2229 using acetate compared to 3.3 ± 0.1 g L^−1^ for its glucose reference, 2.9 ± 0.1 g L^−1^ vs. 2.1 ± 0.0 g L^−1^ for *U. rabenhorstiana* #2708, and 2.9 ± 0.0 g L^−1^ vs. 1.7 ± 0.1 g L^−1^ for *U. cynodontis* #2705. Obtained itaconate concentrations were lower compared to production screening experiments in small-scale 24-deep-well plates which might be explained by process changes due to the upscaling procedure and/or non-optimized process parameters. Compared to the small-scale screening experiments, higher biomass formation was observed during controlled batch fermentation for *U. maydis* #2229 and *U. rabenhorstiana* #2708. *U. maydis* #2229 obtained an OD_600_ of 49 ± 1 in a small scale compared to 59 ± 1 during batch cultivation experiments in addition to acetate. Therefore, decreased itaconate titers could be explained by a higher biomass formation due to, e.g., pH control and better oxygen supply. Three different strains were tested during batch cultivations, and their optimum as far as pH, air supply, buffer system, and carbon source ratio might be different among the strains.

As far as *U. rabenhorstiana* #2708 is concerned, itaconate production was lower compared to published data [57]. The highest itaconate titer of 31.7 g L^−1^ reported was reached in a batch fermentation with 100 g L^−1^ glucose at pH 6.0, corresponding to a yield of 0.34 (*w/w*) [58]. Comparing the process parameter, oxygen supply is a critical factor. During this study, DOT was kept constant at 80% (aeration cascade 800–1200 rpm, 2 vvm). In contrast, Krull et al. observed that the best results were achieved for itaconic acid production with *U. rabenhorstiana* at the lowest aeration rate of 0.1 vvm and a constant stirring rate of 500 rpm regarding titer, productivity, and yield [57]. Furthermore, they observed that the increase in aeration and stirring rate was related to the formation of 36% more biomass at higher aeration rates because of a better supply of oxygen. Furthermore, several pH values were tested during a study performed by Krull et al. Increasing the pH to 6.5 leads to a decrease in itaconate production. Thus, bioprocess development should be proceeded to enhance itaconate production of the respective Ustilaginaceae candidates during subsequent experiments.

**Figure 7:**
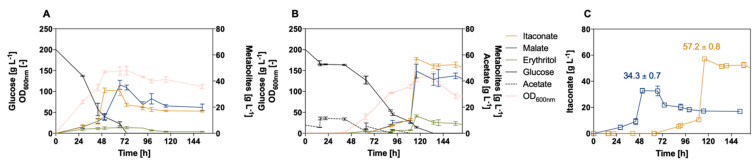
Controlled-batch fermentations of *U. maydis* #2229 increasing glucose concentration to 200 g L^−1^. (**A**) Bioreactor experiments using 200 g L^−1^ glucose, (**B**) with 200 g L^−1^ glucose and 25 g L^−1^ acetate. Cultivations were performed in MTM medium (0.8 g L^−1^ NH_4_Cl, 30 °C, 80% DOT, at pH 6.5). (**C**) Overview of itaconate production of both experiments. Cultivations using acetate as a co-substrate are shown in orange (Y_P/S_ = 0.25 ± 0.00 [g_ITA_/g_c-source_]), and respective glucose references are shown in blue (Y_P/S_ = 0.17 ± 0.00 [g_ITA_/g_c-source_]). Error bars indicate the standard error of the mean (*n* = 3).

The batch fermentations of *U. maydis* #2229 confirmed the product range found in the screening approaches for glucose, although published itaconate concentrations produced under similar conditions could not be reached [55]. Therefore, additional bioreactor experiments were performed with *U. maydis* #2229, increasing the glucose concentration to 200 g L^−1^ and 25 g L^−1^ acetate accordingly (Figure 7).

Those cultivations led to an itaconate titer of 57.2 ± 0.8 g L^−1^ in the presence of the co-substrate compared to 34.3 ± 0.7 g L^−1^ itaconate of the glucose reference. The yield was improved to 0.25 ± 0.00 [Y_P/S_ *=* g_ITA_/g_c-source_]. Maassen et al. obtained a similar itaconate titer of 32.6 ± 0.8 g L^−1^ using 200 g L^−1^ glucose [55]. The *U. cynodontis* #2705 wildtype can only be compared to previous studies with *U. cynodontis* #2706 reaching titers around 5 g L^−1^ itaconate using 50 g L^−1^ glucose [17].

### 3.4. Extracellular Metabolite Identification via LC-UV/RI-MS/MS

**Figure 8:**
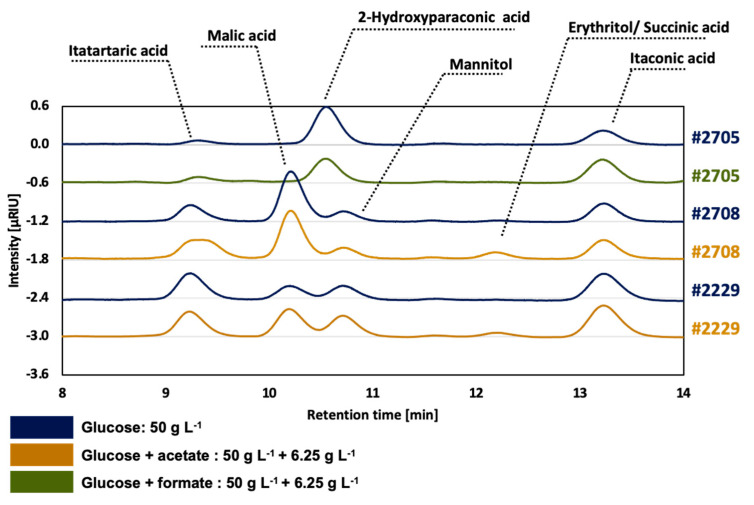
HPLC chromatogram overlay (RI detector). Extracellular metabolites in the supernatant formed by selected Ustilaginaceae candidates. Samples were taken after 75, 64, and 114 h of *U. maydis* #2229, *U. rabenhorstiana* #2708, and *U. cynodontis* #2705, respectively, during fermentation in a bioreactor containing MTM medium (0.8 g L^−1^ NH_4_Cl, 30 °C, 80% DOT, at pH 6.5). Ustilaginaceae using acetate as a co-substrate are shown in orange; formate co-substrate cultivations are shown in green (6.25 g L^−1^). Respective glucose references (50 g L^−1^) are shown in blue.

A recent study from Becker et al. showed that *U. maydis* chassis strain development leads to an increased itaconate titer due to a reduced by-product spectrum [23]. Ustilaginaceae are known to show a versatile product spectrum including organic acids (e.g., itaconate, malate, succinate), polyols (e.g., erythritol, mannitol), and extracellular glycolipids [15,16,58,59,60,61]. Thus, metabolic engineering and by-product reduction display a promising strategy for chassis strain development. To identify interesting targets for metabolic engineering, extracellular metabolites of the three selected strains were analyzed during this study. Extracellular metabolites were identified via LC-UV/RI-MS/MS while MS/MS mass spectrums of samples were compared directly to authentic standards and are displayed in Figures S3 and S4. Identification of metabolites such as malate, itatartarate, 2-hydroxyparaconic acid, mannitol, erythritol, succinate, and itaconic acid was carried out and implemented in the established HPLC method. Figure 8 displays an HPLC chromatogram overlay incorporating the identified metabolites obtained during controlled batch fermentations. Thereby, the diversity in extracellular metabolites between the different tested strains *U. maydis* #2229, *U. rabenhorstiana* #2708, and *U. cynodontis* #2705 was observed. Representative time points were chosen for each strain and condition where the highest number of peaks was detected. Thus, analyzed samples were taken after 75, 64, and 114 h during the fermentation of *U. maydis* #2229, *U. rabenhorstiana* #2708, and *U. cynodontis* #2705, respectively.

Thereby, differences between the tested strains and each condition were observed. Besides itaconic acid, the major extracellular metabolite produced by *U. cynodontis* #2705 was 2-hydroxyparaconic acid. In contrast, *U. maydis* and *U. rabenhorstiana* showed quite different extracellular metabolic profiles as significant mannitol as well as malic acid production was observed (Figure S5). When acetate was added to the medium, these strains exhibited higher erythritol production comparing to the control condition without acetate. Itatartarate and itaconate were identified in all of the displayed samples.

Based on the shown extracellular metabolites results as well as recent studies, several options for metabolic engineering can be employed to alter the metabolic flux distribution to maximize product synthesis. Potential targets are, e.g., overexpression of the mitochondrial transporter Mtt1 [23], the overexpression of the cluster-associated regulator Ria1, disrupting the itaconate oxidase encoding gene *cyp3*, reducing by-product spectrum of extracellular glycolipids as well as heterologous expression of the mitochondrial transporter MttA from *A. terreus* [2,23]. Furthermore, deletion of *fuz7* enables a stable yeast-like growth [17]. Moreover, a metabolomics method focusing on the central carbon metabolism has recently been developed for *U. maydis*, which can be applied to investigate the cellular metabolic network and support metabolic engineering strategy [62].

## 4. Conclusions

Here, we report the co-utilization of acetate and formate by strains of the genus Ustilaginaceae. From 72 different Ustilaginaceae strains of 36 species, *U. maydis* MB215 (#2229) and *U. rabenhorstiana* NBRC 8995 (#2708) were identified as promising candidates for acetate co-metabolization while *U. cynodontis* NBRC 7530 (#2705) was identified as a potential production host using formate as a co-substrate for the production of itaconate, a platform chemical for polymer and biofuel production. The current industrial production of plastic monomers and fuels from fossil resources has to be reduced and in the long-run stopped, requiring alternative technologies. Itaconate is promising, as it has been, for the last 70 years, produced in fermentations using sugars as substrate. However, with a shift of the carbon source in the chemical industry, land-use for sugar production for biotechnology would be skyrocketing, a scenario that will come fast to a maximum, although most agricultural land is still used for meat production, and only about 25% of all grains are used for human consumption. Still, the use of carbon sources that are derived from CO_2_ and green hydrogen opens possibilities for the carbon-neutral production of chemicals and fuels and, most importantly, scales without a proportional land-use.

While acetate can be directly used as a carbon and energy source, formate co-consumption only delivers extra electrons to the fungal metabolism. The co-substrate strategies presented here indeed highlighted single strains of the Ustilaginaceae that could not only utilize simultaneously both substrates but also produce more itaconate. Nevertheless, individual bioprocess development is essential to further improve itaconate production and evaluate their capabilities. During this study, the tested wildtype strains produced a broad range of extracellular products, emphasizing the biodiversity of this microbial family. Based on the shown data on extracellular metabolites and previous results, several options for metabolic engineering were displayed to alter the metabolic flux distribution to maximize product synthesis. As far as co-substrate utilization is concerned, an adaptive laboratory is a valuable tool potentially enhancing co-substrate tolerance and utilization. Furthermore, the optimum glucose co-substrate ratio will be determined during subsequent Design of Experiment (DoE) approaches enabling the development of a suitable co-feeding strategy. Specifically, C^13^-labelling experiments can lead to a better understanding of acetate and formate assimilation pathways in Ustilaginaceae contributing toward a carbon-neutral itaconate production in the future. These efforts will showcase the reduction of the carbon footprint of biotechnology, without increasing land-use. The latter not only is, in the authors’ opinion, a major driver for the acceptance of the transition in the chemical industry from fossil to renewable carbon sources but also opens up opportunities for stabilizing soil and water health and thereby biodiversity.

## Figures and Tables

**Table 1:** Production parameters of controlled-batch fermentations of *U. maydis* #2229, *U. rabenhorstiana* #2708, and *U. cynodontis* #2705. Fermentation experiments were performed in a bioreactor containing MTM medium with 50 g L^−1^ glucose, 0.8 g L^−1^ NH_4_Cl, at 30 °C, 80% DOT, and pH 6.5. Co-substrates (acetate, formate) were added with 6.25 g L^−1^. Statistically significant differences in itaconic acid production (*p* ≤ 0.05) are indicated as *. Details of statistical analyses are displayed in Table S11.

Strain	C-Source	Titer_max_ [g L^−1^]	Y_P/S_ [g_ITA_/g_c-source_]	Y_P/S_ [C-moL_ITA_/C-moL_c-source_]
*U. maydis* #2229	Glucose	3.2	0.07	0.08
		3.3	0.07	0.08
	Glucose + Acetate	4.9	0.09	0.10
		4.6	0.08	0.10
*U. rabenhorstiana* #2708	Glucose	2.1	0.04	0.05
		2.1	0.04	0.05
	Glucose + Acetate	2.7	0.05	0.06
		3.1	0.06	0.07
*U. cynodontis* #2705 *	Glucose	1.7	0.03	0.04
		1.6	0.03	0.04
	Glucose + Formate	2.9	0.05	0.06
		2.9	0.05	0.06

## Supplementary Materials

The following are available online at https://www.mdpi.com/2309-608X/7/2/98/s1, Figure S1: Itaconic acid production of most promising Ustilaginaceae strains using different carbon source concentrations, Figure S2: C-source consumption during controlled-batch fermentations of selected Ustilaginaceae candidates, Figure S3: Extracellular metabolite identification via LC-UV/RI-MS/MS, Figure S4: MS/MS spectrum of ITT, Figure S5: Malate production during controlled-batch fermentations of selected Ustilaginaceae candidates, Table S1: Ustilaginaceae strains screened in this study, Table S2: Maximum optical densities (OD [−]) of all 72 strains obtained during biodiversity screening, Table S3: Maximum growth rates (µ_max_ [h^−1^]) of all 72 strains obtained during biodiversity screening, Table S4: pH [−] values of all 72 strains obtained during biodiversity screening, Table S5: Production parameters of biodiversity screening for acetate co-utilization, Table S6: Production parameters of biodiversity screening for formate co-utilization, Table S7: Production parameters of System Duetz cultivation for acetate co-utilization, Table S8: Production parameters of System Duetz cultivation for formate co-utilization, Table S9: pH values during System Duetz cultivation for acetate co-utilization, Table S10: pH values during System Duetz cultivation for formate co-utilization, Table S11: Welch *t*-test results obtained for *U. maydis* #2229, *U. rabenhorstiana* #2708, and *U. cynodontis* #2705 during small-scale production and bioreactor experiments, Table S12: Welch t-test results obtained during small-scale production experiments.

## References

[1] Okabe M., Lies D., Kanamasa S., Park E.Y. (2009). Biotechnological production of itaconic acid and its biosynthesis in *Aspergillus terreus*. Appl. Microbiol. Biotechnol..

[2] Hosseinpour Tehrani H., Becker J., Bator I., Saur K., Meyer S., Rodrigues Lóia A.C., Blank L.M., Wierckx N. (2019). Integrated strain- and process design enable production of 220 g L^−1^ itaconic acid with *Ustilago maydis*. Biotechnol. Biofuels.

[3] Robert T., Friebel S. (2016). Itaconic acid—A versatile building block for renewable polyesters with enhanced functionality. Green Chem..

[4] Steiger M.G., Wierckx N., Blank L.M., Mattanovich D., Sauer M. (2016). Itaconic acid an emerging building block. Industrial Biotechnology: Products and Processes.

[5] Klement T., Büchs J. (2013). Itaconic acid—a biotechnological process in change. Bioresour. Technol..

[6] Kumar S., Krishnan S., Samal S.K., Mohanty S., Nayak S.K. (2017). Itaconic acid used as a versatile building block for the synthesis of renewable resource-based resins and polyesters for future prospective: A review. Polym. Int..

[7] Saha B.C. (2017). Emerging biotechnologies for production of itaconic acid and its applications as a platform chemical. J. Ind. Microbiol. Biot..

[8] Willke T., Vorlop K.D. (2001). Biotechnological production of itaconic acid. Appl. Microbiol. Biot..

[9] Geilen F.M., Engendahl B., Harwardt A., Marquardt W., Klankermayer J., Leitner W. (2010). Selective and flexible transformation of biomass-derived platform chemicals by a multifunctional catalytic system. Angew. Chem. Int. Ed. Engl..

[10] Kuenz A., Krull S. (2018). Biotechnological production of itaconic acid—Things you have to know. Appl. Microbiol. Biotechnol..

[11] Karaffa L., Diaz R., Papp B., Fekete E., Sandor E., Kubicek C.P. (2015). A deficiency of manganese ions in the presence of high sugar concentrations is the critical parameter for achieving high yields of itaconic acid by *Aspergillus terreus*. Appl. Microbiol. Biotechnol..

[12] Krull S., Hevekerl A., Kuenz A., Prüße U. (2017). Process development of itaconic acid production by a natural wild type strain of *Aspergillus terreus* to reach industrially relevant final titers. Appl. Microbiol. Biotechnol..

[13] Gyamerah M. (1995). Factors affecting the growth form of *Aspergillus terreus* NRRL 1960 in relation to itaconic acid fermentation. Appl. Microbiol. Biotechnol..

[14] Karaffa L., Kubicek C.P. (2019). Citric acid and itaconic acid accumulation: Variations of the same story?. Appl. Microbiol. Biotechnol..

[15] Geiser E., Wiebach V., Wierckx N., Blank L.M. (2014). Prospecting the biodiversity of the fungal family Ustilaginaceae for the production of value-added chemicals. Fungal Biol. Biotechnol..

[16] Guevarra E.D., Tabuchi T. (1990). Accumulation of itaconic, 2-hydroxyparaconic, itatartaric, and malic-acids by strains of the genus Ustilago. Agric. Biol. Chem..

[17] Hosseinpour Tehrani H., Tharmasothirajan A., Track E., Blank L.M., Wierckx N. (2019). Engineering the morphology and metabolism of pH tolerant *Ustilago cynodontis* for efficient itaconic acid production. Metab. Eng..

[18] Zambanini T., Hosseinpour Tehrani H., Geiser E., Merker D., Schleese S., Krabbe J., Buescher J.M., Meurer G., Wierckx N., Blank L.M. (2017). Efficient itaconic acid production from glycerol with *Ustilago vetiveriae* TZ1. Biotechnol. Biofuels.

[19] Geiser E., Tehrani H.H., Meyer S., Blank L.M., Wierckx N. (2018). Evolutionary freedom in the regulation of the conserved itaconate cluster by Ria1 in related Ustilaginaceae. Fungal Biol. Biotechnol..

[20] Geiser E., Przybilla S.K., Engel M., Kleineberg W., Buttner L., Sarikaya E., Den Hartog T., Klankermayer J., Leitner W., Bölker M. (2016). Genetic and biochemical insights into the itaconate pathway of *Ustilago maydis* enable enhanced production. Metab. Eng..

[21] Steinberg G., Perez-Martin J. (2008). *Ustilago maydis,* a new fungal model system for cell biology. Trends. Cell. Biol..

[22] Vollmeister E., Schipper K., Feldbrügge M. (2012). Microtubule-dependent mRNA transport in the model microorganism *Ustilago maydis*. RNA Biol..

[23] Becker J., Hosseinpour Tehrani H., Gauert M., Mampel J., Blank L.M., Wierckx N. (2019). An *Ustilago maydis* chassis for itaconic acid production without by-products. Microb. Biotechnol..

[24] Weastra (2013). WP 8.1. Determination of Market Potential for Selected Platform Chemicals: Itaconic Acid, Succinic acid, 2,5-furandicarboxylic Acid.

[25] Bafana R., Pandey R.A. (2018). New approaches for itaconic acid production: Bottlenecks and possible remedies. Crit. Rev. Biotechnol..

[26] Blank L.M., Narancic T., Mampel J., Tiso T., O’Connor K. (2020). Biotechnological upcycling of plastic waste and other non-conventional feedstocks in a circular economy. Curr. Opin. Biotechnol..

[27] Kolláth I.S., Molnár Á.P., Soós Á., Fekete E., Sándor E., Kovács B., Kubicek C.P., Karaffa L. (2019). Manganese Deficiency Is Required for High Itaconic Acid Production from D-Xylose in *Aspergillus terreus*. Front. Microbiol..

[28] Westbrook A.W., Miscevic D., Kilpatrick S., Bruder M.R., MooYoung M., Chou C.P. (2019). Strain engineering for microbial production of value-added chemicals and fuels from glycerol. Biotechnol. Adv..

[29] Rohmann K., Kothe J., Haenel M.W., Englert U., Holscher M., Leitner W. (2016). Hydrogenation of CO_2_ to formic acid with a highly active ruthenium acriphos complex in DMSO and DMSO/ water. Angew. Chem. Int. Ed..

[30] Groher A., Weuster-Botz D. (2016). Comparative reaction engineering analysis of different acetogenic bacteria for gas fermentation. J. Biotechnol..

[31] Park J.O., Liu N., Holinski K.M., Emerson D.F., Qiao K.J., Woolston B.M., Wu J., Lazar Z., Islam M.A., Vidoudez C. (2019). Synergistic substrate cofeeding stimulates reductive metabolism. Nat. Metab..

[32] Gildemyn S., Verbeeck K., Slabbink R., Andersen S.J., Prévoteau A., Rabaey K. (2015). Integrated Production, Extraction, and Concentration of Acetic Acid from CO_2_ through Microbial Electrosynthesis. Environ. Sci. Technol. Lett..

[33] Nielsen J. (2014). Synthetic biology for engineering acetyl coenzyme A metabolism in yeast. mBio.

[34] Turcotte B., Liang X.B., Robert F., Soontorngun N. (2010). Transcriptional regulation of nonfermentable carbon utilization in budding yeast. FEMS Yeast Res..

[35] Vilela-Moura A., Schuller D., Mendes-Faia A., Silva R.D., Chaves S.R., Sousa M.J., Corte-Real M. (2011). The impact of acetate metabolism on yeast fermentative performance and wine quality: Reduction of volatile acidity of grape musts and wines. Appl. Microbiol. Biotechnol..

[36] Hargreaves J.A., Turner G. (1989). Isolation of the Acetyl-CoA Synthase Gene from the Corn Smut Pathogen *Ustilago maydis*. J. Gen. Microbiol..

[37] Kretschmer M., Lambie S., Croll D., Kronstad J.W. (2018). Acetate provokes mitochondrial stress and cell death in *Ustilago maydis*. Mol. Microbiol..

[38] Geiser E., Przybilla S.K., Friedrich A., Buckel W., Wierckx N., Blank L.M., Bölker M. (2016). Itaconic acid biosynthesis in *U. maydis*. Microb. Biotechnol..

[39] Tishkov V.I., Popov V.O. (2004). Catalytic mechanism and application of formate dehydrogenase. Biochemistry.

[40] (2019). UMAG_05170 Gene & Protein, Formate Dehydrogenase-Ustilago Maydis (Strain 521/FGSC 9021), UniprotKB. A0A0D1C9V6. https://www.uniprot.org/uniprot/A0A0D1C9V6.

[41] Overkamp K.M., Kötter P., van der Hoek R., Schoondermark-Stolk S., Luttik M.A.H., Van Dijken J.P., Pronk J.T. (2002). Functional analysis of structural genes for NAD^+^-dependent formate dehydrogenase in *Saccharomyces cerevisiae*. Yeast.

[42] Gonzalez de la Cruz J., Machens F., Messerschmidt K., Bar-Even A. (2019). Core Catalysis of the Reductive Glycine Pathway Demonstrated in Yeast. ACS Synth. Biol..

[43] Cotton C.A.R., Claassens N.J., Benito-Vaquerizo S., Bar-Even A. (2020). Renewable methanol and formate as microbial feedstocks. Curr. Opin. Biotechnol..

[44] Yishai O., Lindner S.N., Gonzalez de la Cruz J., Tenenboim H., Bar-Even A. (2016). The formate bio-economy. Curr. Opin. Chem. Biol..

[45] Roe A.J., McLaggan D., Davidson I., O’Byrne C., Booth I.R. (1998). Perturbation of anion balance during inhibition of growth of *Escherichia coli* by weak acids. J. Bacteriol..

[46] Russell J.B., Diez-Gonzalez F. (1998). The effects of fermentation acids on bacterial growth. Adv. Microb. Physiol..

[47] Schmidt T., Schaechter M. (2012). Topics in Ecological and Environmental Microbiology.

[48] Enjalbert B., Millard P., Dinclaux M., Portais J.-C., Létisse F. (2017). Acetate fluxes in *Escherichia coli* are determined by the thermodynamic control of the Pta-AckA pathway. Sci. Rep..

[49] Duetz W.A., Rüedi L., Hermann R., O’Connor K., Büchs J., Witholt B. (2000). Methods for Intense Aeration, Growth, Storage, and Replication of Bacterial Strains in Microtiter Plates. Appl. Environ. Microbiol..

[50] Djoumbou-Feunang Y., Pon A., Karu N., Zheng J., Li C., Arndt D., Gautam M., Allen F., Wishart D. (2019). Significantly Improved ESI-MS/MS Prediction and Compound Identification. Metabolites.

[51] Howe E., Holton K., Nair S., Schlauch D., Sinha R., Quackenbush J., Ochs M., Casagrande J., Davuluri R. (2010). MeV: MultiExperiment Viewer. Biomedical Informatics for Cancer Research.

[52] Hemmerich J., Wiechert W., Oldiges M. (2017). Automated growth rate determination in high-throughput microbioreactor systems. BMC Res. Notes.

[53] Salmond C.V., Kroll R.G., Booth I.R. (1984). The effect of food preservatives on pH homeostasis in *Escherichia coli*. J. Gen. Microbiol..

[54] Lastauskienė E., Zinkevičienė A., Girkontaitė I., Kaunietis A., Kvedarienė V. (2014). Formic acid and acetic acid induce a programmed cell death in pathogenic *Candida* species. Current Microbiol..

[55] Maassen N., Panakova M., Wierckx N., Geiser E., Zimmermann M., Klinner U., Blank L.M. (2013). Influence of carbon and nitrogen concentration on itaconic acid production by the smut fungus *Ustilago maydis*. Eng. Life Sci..

[56] Zambanini T., Hartmann S.K., Schmitz L.M., Büttner L., Hosseinpour Tehrani H., Geiser E., Beudels M., Venc D., Wandrey G., Büchs J. (2017). Promoters from the itaconate cluster of *Ustilago maydis* are induced by nitrogen depletion. Fungal Biol. Biotechnol..

[57] Krull S., Lünsmann M., Prüße U., Kuenz A. (2020). *Ustilago Rabenhorstiana*—An Alternative Natural Itaconic Acid Producer. Fermentation.

[58] Zambanini T., Sarikaya E., Kleineberg W., Buescher J.M., Meurer G., Wierckx N., Blank L.M. (2016). Efficient malic acid production from glycerol with *Ustilago trichophora* TZ1. Biotechnol. Biofuels.

[59] Feldbrügge M., Kellner R., Schipper K. (2013). The biotechnological use and potential of plant pathogenic smut fungi. Appl. Microbiol. Biotechnol..

[60] Morita T., Fukuoka T., Imura T., Kitamoto D. (2009). Production of glycolipid biosurfactants by basidiomycetous yeasts. Biotechnol. Appl. Biochem..

[61] Jeya M., Lee K.-M., Tiwari M.K., Kim J.-S., Gunasekaran P., Kim S.-Y., Kim I.-W., Lee J.-K. (2009). Isolation of a novel high erythritol-producing *Pseudozyma tsukubaensis* and scale-up of erythritol fermentation to industrial level. Appl. Microbiol. Biotechnol..

[62] Phan A.N.T., Blank L.M. (2020). GC-MS-Based Metabolomics for the Smut Fungus *Ustilago maydis*: A Comprehensive Method Optimization to Quantify Intracellular Metabolites. Front. Mol. Biosci..

