# Supplementary material for: Ustilaginaceae Biocatalyst for Co-Metabolism of CO2-Derived Substrates toward Carbon-Neutral Itaconate Production
Source: J Fungi (Basel). 2021 Jan 29;7(2):98. doi: 10.3390/jof7020098 (PMC7911105; doi:10.3390/jof7020098)
Supplement: Supplementary file 1 [file jof-07-00098-s001.pdf]

Supplementary materials

Supplementary figures

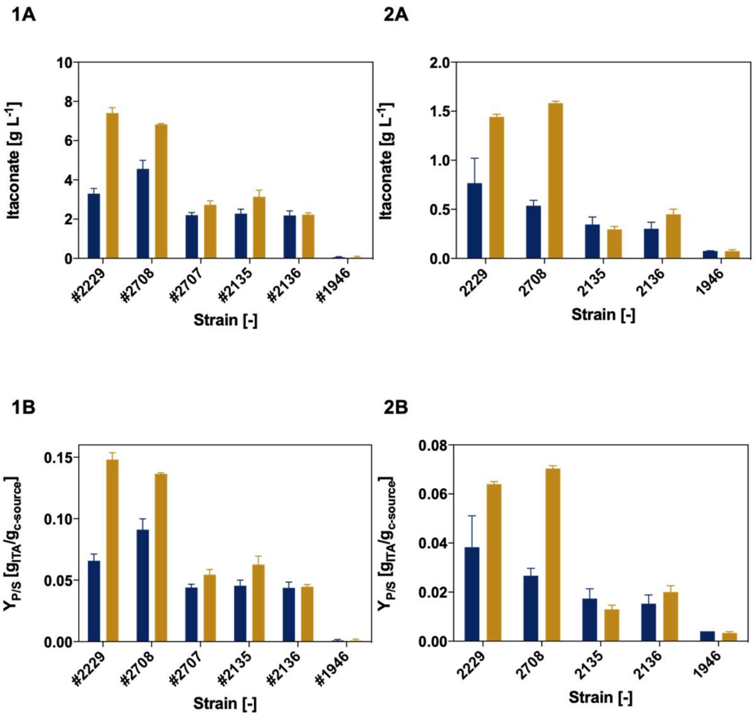

Figure S1 Itaconic acid production of most promising Ustilaginaceae strains using different carbon source concentrations. (A) Maximum itaconic acid production [g L^{-1}] and (B) $Y_{P/S}$ [$g_{ITA}/g_{c\text{-}source}$] during System Duetz® 24-deep well plates cultivation experiments with 1.5 ml MTM medium and 0.8 g L^{-1} NH_4Cl. Cultivations were performed with two different carbon-source concentrations (1) 50 g L^{-1} glucose + 6.25 g L^{-1} acetate and (2) 20 g L^{-1} glucose + 2.5 g L^{-1} acetate. Ustilaginaceae candidates using acetate as a co-substrate are shown in orange, respective glucose references are shown in blue. Error bars indicate the deviation from the mean for n=2.

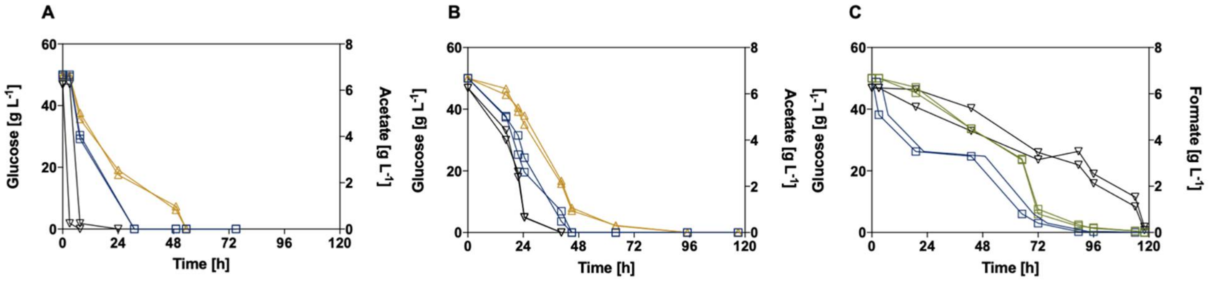

Figure S2 C-source consumption during controlled batch fermentations of selected Ustilaginaceae candidates. (A) *U. maydis* #2229, (B) *U. rabenhorstiana* #2708 and (C) *U. cynodontis* #2705 fermentation in a bioreactor containing MTM medium (0.8 g L^{-1} NH_4Cl, 30°C, 80% DOT, at pH 6.5). Glucose consumptions using acetate as a co-substrate are shown in orange, formate co-substrate cultivations are shown in green. Respective glucose references (50 g L^{-1}) are shown in blue. Black indicates co-substrate consumption (6.25 g L^{-1}).

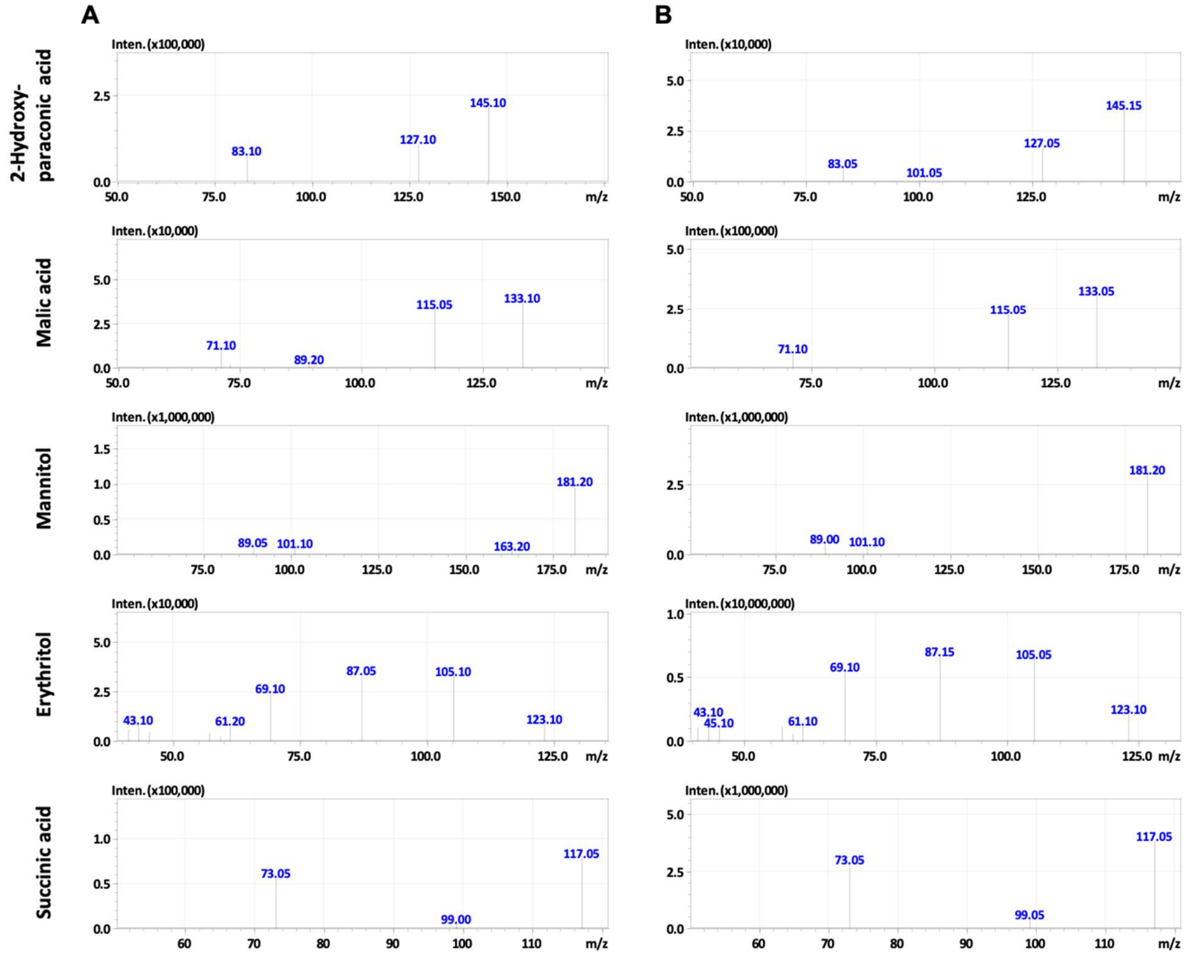

Figure S3 Extracellular metabolite identification via LC-UV/RI-MS/MS. (A) Product ion scan for extracellular metabolite samples and (B) their corresponding standards. Extracellular metabolites in the supernatant formed by *U. maydis* #2229, *U. rabenhorstiana* #2708, and *U. cynodontis* #2705. Samples were taken after 75, 64, and 114 h during fermentation in a bioreactor containing MTM medium (0.8 g L $^{-1}$ NH_4Cl, 19.5 g L^{-1} MES, 30°C, 80% DOT, at pH 6.5).

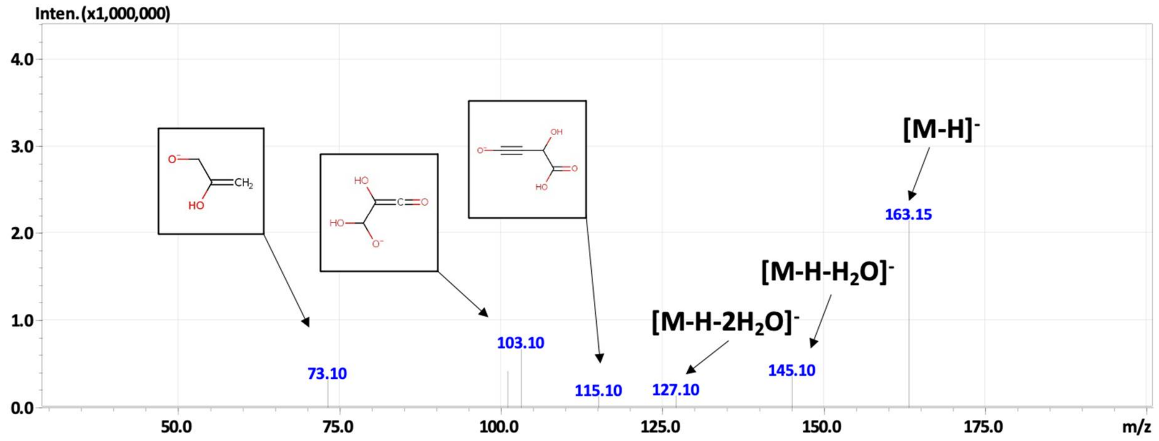

Figure S4 MS/MS spectrum of ITT. Structure prediction of itatartarate (ITT) was performed via CFM-ID 3.0 [1].

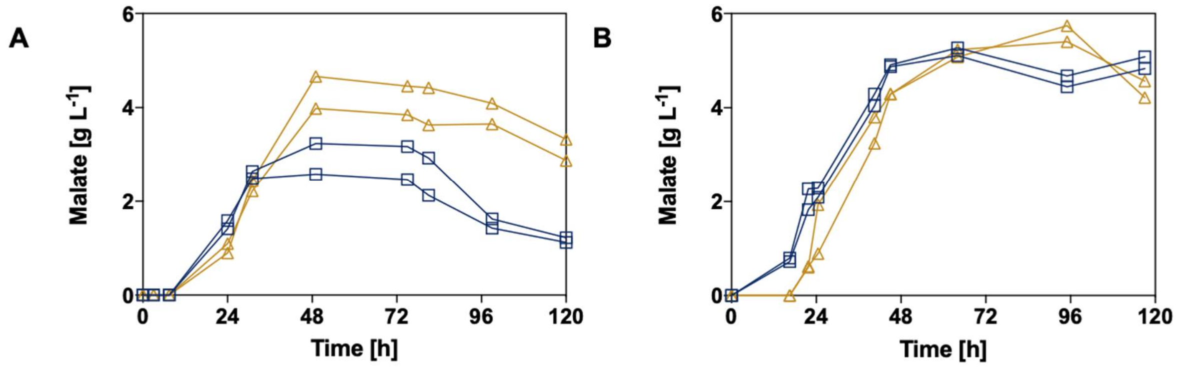

Figure S5 Malate production during controlled batch fermentations of selected Ustilaginaceae candidates. (A) *U. maydis* #2229 and (B) *U. rabenhorstiana* #2708 fermentation in a bioreactor containing MTM medium (0.8 g L^{-1} NH_4Cl, 30°C, 80% DOT, at pH 6.5). Glucose consumptions using acetate as a co-substrate are shown in orange. Respective glucose references (50 g L^{-1}) are shown in blue. Black indicates co-substrate consumption (6.25 g L^{-1}).

Supplementary tables

Table S1 Ustilaginaceae strains screened in this study.

No.	Strain		Origin
#1946	*Pseudozyma antartica*		NRRL Y – 7808
#1947	*Pseudozyma antartica*		NRRL Y - 8295
#1949	*Ustilago maydis*		DSM 3121
#1951	*Ustilago maydis*		DSM 14603
#2134	*Ustilago maydis*	Nr. 196	Prof. M. Bölker, Philipps University Marburg, Germany
#2135	*Ustilago maydis*	Nr. 197	
#2136	*Ustilago maydis*	Nr. 198	
#2144	*Ustilago maydis*	Nr. 206	
#2152	*Ustilago maydis*	Nr. 215	
#2158	*Ustilago maydis*	Nr. 469	
#2162	*Ustilago maydis*	Nr. 474	
#2167	*Ustilago maydis*	Nr. 480	
#2169	*Ustilago maydis*	Nr. 482	
#2172	*Ustilago maydis*	Nr. 485	
#2177	*Ustilago maydis*	Nr. 491	
#2178	*Ustilago maydis*	Nr. 492	
#2179	*Ustilago maydis*	Nr. 495	
#2196	*Ustilago maydis*	FB1 Mating type a1b1	Banuett & Herskowitz, 1989, Minnesoty USA
#2197	*Ustilago maydis*	FB2 Mating type a2b2	
#2199	*Ustilago maydis*	RK 123	Ronny Kellner [2]
#2205	*Ustilago maydis*	RK 212	
#2208	*Ustilago maydis*	RK215	
#2209	*Macalpinomyces eriachnes*	RK 028	CBS 131454
#2210	*Sporisorium consanguineum*	RK 133	CBS 131454
#2211	*Sporisorium cruentum*	UMa920, Mating type MAT1	CBS 131456
#2212	*Sporisorium exsertum*	RK 033	CBS 133249
#2213	*Sporisorium scitamineum*	UMa698, Sscl4, JS109, RK 109, Mating type MAT1	CBS 131457

#2214	*Sporisorium walkeri*	RK 031	CBS 131462
#2215	*Ustanciosporium gigantosporum*	UMa706	CBS 131464
#2218	*Ustilago filiformis*	UMa701	CBS 131469
#2219	*Ustilago trichophora*	RK 089	CBS 131473
#2220	*Ustilago vetiveriae*	RK 075	CBS 131474
#2221	*Ustilago xerochloae*	UMa702	CBS 131476
#2229	*Ustilago maydis*	MB215	DSM 17144
#2696	*Pseudozyma hubeiensis*		NBRC 105053
#2697	*Pseudozyma hubeiensis*		NBRC 105054
#2698	*Pseudozyma hubeiensis*		NBRC 105055
#2699	*Ustilago trichophora*		NBRC 100155
#2700	*Ustilago trichophora*		NBRC 100156
#2701	*Ustilago trichophora*		NBRC 100157
#2702	*Ustilago trichophora*		NBRC 100158
#2703	*Ustilago trichophora*		NBRC 100159
#2704	*Ustilago trichophora*		NBRC 100160
#2705	*Ustilago cynodontis*		NBRC 7530
#2706	*Ustilago cynodontis*		NBRC 9727
#2707	*Ustilago cynodontis*		NBRC 9758
#2708	*Ustilago rabenhorstiana*		NBRC 8995
#2710	*Pseudozyma tsukubaensis*		NBRC 1940
#2813	*Sporisorium lanigeri*	BRIP 27609 a	Queensland Plant Pathology Herbarium, Australia
#2814	*Macalpinomyces mackinlayi*	BRIP 52549 a	
#2815	*Sporisorium cenchri-elymoidis*	BRIP 26491 a	
#2816	*Macalpinomyces ordensis*	BRIP 26904 a	
#2817	*Ustilago schmidtiae*	BRIP 26906 a	
#2818	*Sporisorium bothriochloae*	BRIP 26908 a	
#2819	*Sporisorium themedae*	BRIP 26917 a	
#2820	*Sporisorium tumiforme*	BRIP 26919 a	
#2821	*Ustilago curta*	BRIP 26929 a	
#2822	*Ustilago triodiae*	BRIP 26907 a	

#2823	*Sporisorium setariae*	BRIP 26910 a	
#2824	*Ustilago cynodontis*	BRIP 28040 a	
#2825	*Sporisorium caledonicum*	BRIP 28043 a	
#2826	*Ustilago lituana*	BRIP 46795 a	
#2832	*Sporisorium iseilematis-ciliati*	BRIP 60429 a	
#2833	*Macalpinomyces spermophorus*	BRIP 60430 a	
#2834	*Macalpinomyces tubiformis*	BRIP 60434 a	
#2835	*Macalpinomyces spermophorus*	BRIP 60448 a	
#2836	*Ustilago xerochloae*	BRIP 60876 a	
#2838	*Sporisorium iseilematis-ciliati*	BRIP 60887 a	
#2841	*Anthracocystis heteropogonicola*	BRIP 60896 a	
#2842	*Anthracocystis bothriochloae*	BRIP 60901 a	
#2850	*Ustilago egenula*	BRIP 60884 a	
#3600	*Ustilago maydis*		Bernd Leuchtle [3]

Table S2 Maximum optical densities (OD [-]) of all 72 strains obtained during biodiversity screening. Whole set of growth screening results using the Growth Profiler device by Enzyscreen with different acetate and formate concentrations as co-substrates in combination with 20 g L^{-1} glucose (n=2).

Strain	Glucose	2.5 g L^{-1} Acetate	5 g L^{-1} Acetate	10 g L^{-1} Acetate	2.5 g L^{-1} Formate	5 g L^{-1} Formate	10 g L^{-1} Formate
#1946	46.8 ± 0.6	55.9 ± 1.5	47.4 ± 0.3	46.4 ± 0.1	50.0 ± 0.7	42.5 ± 0.1	35.1 ± 0.2
#1947	37.0 ± 1.1	45.0 ± 0.1	42.5 ± 0.0	33.2 ± 0.6	40.8 ± 0.1	34.3 ± 1.5	27.8 ± 0.1
#1949	44.4 ± 0.5	55.5 ± 1.0	35.2 ± 0.1	52.9 ± 0.3	19.2 ± 0.2	21.2 ± 0.1	4.1 ± 0.5
#1951	34.3 ± 1.4	52.3 ± 1.6	43.8 ± 0.1	49.1 ± 0.3	21.4 ± 0.4	19.3 ± 0.1	17.5 ± 1.9
#2134	32.4 ± 0.3	27.3 ± 0.6	22.8 ± 0.6	38.8 ± 2.3	24.5 ± 2.7	22.6 ± 0.3	23.5 ± 2.2
#2135	19.7 ± 2.8	37.2 ± 0.8	38.2 ± 0.2	49.5 ± 0.4	13.3 ± 1.3	15.3 ± 1.6	18.2 ± 0.9
#2136	29.9 ± 0.1	41.9 ± 1.7	44.6 ± 0.1	51.4 ± 1.5	18.0 ± 0.9	19.4 ± 0.4	18.8 ± 0.5
#2144	33.4 ± 1.1	36.8 ± 1.5	41.6 ± 1.3	42.8 ± 0.9	26.9 ± 0.8	21.5 ± 1.3	24.5 ± 0.9
#2152	34.0 ± 2.8	36.8 ± 1.1	43.2 ± 0.1	10.2 ± 0.3	27.4 ± 0.1	15.9 ± 1.0	23.9 ± 1.1
#2158	41.9 ± 0.3	28.6 ± 1.1	25.0 ± 0.1	50.0 ± 0.6	28.6 ± 0.6	23.2 ± 0.4	22.8 ± 0.2
#2162	27.9 ± 0.4	35.9 ± 0.3	35.4 ± 0.1	30.3 ± 0.2	29.1 ± 3.6	11.2 ± 3.4	21.5 ± 2.1
#2167	37.1 ± 0.1	48.3 ± 0.5	33.6 ± 1.1	44.2 ± 0.3	41.1 ± 0.5	38.8 ± 0.7	30.4 ± 0.8
#2169	25.9 ± 0.5	34.1 ± 0.9	26.8 ± 1.0	27.8 ± 0.1	34.0 ± 1.1	28.8 ± 2.5	23.5 ± 0.4
#2172	26.4 ± 0.2	37.0 ± 0.2	28.7 ± 0.2	33.4 ± 0.2	34.2 ± 0.2	15.8 ± 0.3	25.8 ± 0.5
#2177	29.0 ± 1.2	37.6 ± 0.1	19.8 ± 0.1	40.6 ± 0.1	42.0 ± 1.1	21.4 ± 0.4	19.9 ± 0.1
#2178	24.9 ± 0.1	35.7 ± 0.4	37.9 ± 1.3	0.1 ± 0.0	20.3 ± 1.3	17.2 ± 0.1	2.0 ± 0.1
#2179	27.4 ± 0.1	33.8 ± 0.7	18.8 ± 0.1	28.6 ± 0.1	39.0 ± 0.1	17.9 ± 1.3	20.6 ± 0.4
#2196	36.3 ± 1.5	48.5 ± 1.0	32.6 ± 0.2	38.1 ± 0.2	42.3 ± 1.1	35.0 ± 0.0	30.5 ± 0.4
#2197	29.0 ± 1.3	36.6 ± 1.3	31.0 ± 0.0	30.7 ± 2.0	29.8 ± 1.0	30.9 ± 1.3	27.6 ± 0.1
#2199	36.0 ± 0.6	34.1 ± 1.1	37.4 ± 1.7	41.6 ± 0.6	29.6 ± 0.7	22.8 ± 2.0	24.8 ± 0.6
#2205	19.1 ± 3.0	24.2 ± 2.2	22.6 ± 0.7	4.8 ± 0.5	24.2 ± 2.2	8.2 ± 2.1	10.0 ± 3.6
#2208	18.4 ± 0.5	26.5 ± 0.1	20.6 ± 0.7	3.7 ± 0.2	17.2 ± 0.0	19.6 ± 0.2	13.0 ± 0.4
#2209	31.3 ± 1.1	28.6 ± 0.1	24.2 ± 1.2	6.6 ± 0.1	26.4 ± 0.3	16.2 ± 0.5	16.8 ± 0.1
#2210	35.8 ± 0.5	14.1 ± 0.4	37.4 ± 0.5	2.7 ± 0.2	3.6 ± 0.1	33.3 ± 0.7	23.3 ± 0.0
#2211	16.9 ± 0.5	23.6 ± 0.1	19.7 ± 0.4	32.0 ± 1.0	17.3 ± 0.3	12.1 ± 0.0	30.3 ± 0.6
#2212	38.1 ± 0.6	49.7 ± 0.1	39.3 ± 1.5	18.5 ± 3.0	35.5 ± 2.6	11.0 ± 2.6	23.2 ± 2.2
#2213	32.3 ± 0.3	35.6 ± 0.0	17.3 ± 0.1	0.24 ± 0.0	29.5 ± 0.9	13.3 ± 0.6	18.6 ± 0.4
#2214	38.8 ± 1.3	48.8 ± 1.8	19.7 ± 0.1	8.8 ± 1.0	33.2 ± 0.0	18.4 ± 0.4	34.2 ± 4.3
#2215	47.2 ± 0.6	42.2 ± 0.1	50.8 ± 0.2	18.6 ± 2.5	37.7 ± 2.8	30.2 ± 0.0	33.9 ± 0.2
#2218	30.6 ± 0.6	38.6 ± 2.2	17.5 ± 0.4	0.6 ± 0.3	30.6 ± 0.5	15.9 ± 0.3	12.0 ± 0.0
#2219	38.2 ± 0.0	35.3 ± 0.0	15.2 ± 1.6	25.3 ± 4.5	39.5 ± 0.0	16.1 ± 2.0	26.4 ± 0.2
#2220	41.3 ± 0.9	49.9 ± 0.1	27.3 ± 0.1	26.3 ± 1.1	34.7 ± 0.1	14.6 ± 0.7	1.1 ± 0.6
#2221	37.7 ± 0.3	42.0 ± 1.6	19.6 ± 0.3	12.4 ± 1.8	30.7 ± 0.4	14.0 ± 0.3	33.0 ± 0.0
#2229	33.2 ± 0.4	33.3 ± 0.0	18.2 ± 1.1	29.9 ± 1.3	28.2 ± 0.4	12.5 ± 1.3	32.6 ± 0.5
#2696	51.7 ± 1.8	59.7 ± 0.1	28.3 ± 0.4	49.2 ± 2.0	47.7 ± 0.0	20.7 ± 0.7	49.2 ± 0.9
#2697	37.0 ± 1.0	53.4 ± 0.1	38.6 ± 0.8	43.5 ± 0.2	56.6 ± 0.8	39.6 ± 0.7	45.2 ± 0.1
#2698	62.2 ± 1.8	72.9 ± 2.2	33.5 ± 0.3	51.8 ± 1.9	61.2 ±1.5	26.0 ± 0.3	49.1 ± 1.0
#2699	22.7 ± 0.8	27.3 ± 1.4	20.7 ± 0.5	30.8 ± 0.4	40.2 ± 0.2	23.6 ± 0.4	22.1 ± 0.0
#2700	28.4 ± 0.0	26.8 ± 3.2	21.3 ± 2.3	36.3 ± 4.9	34.7 ± 4.2	25.1 ± 2.9	22.8 ± 2.7
#2701	35.5 ± 0.5	38.1 ± 0.2	27.1 ± 0.9	47.5 ± 0.1	41.4 ± 3.0	25.8 ± 0.5	25.5 ± 0.2
#2702	22.2 ± 0.9	23.4 ± 0.4	15.7 ± 0.0	34.0 ± 1.1	30.0 ± 0.0	17.4 ± 0.1	16.5 ± 0.8
#2703	47.8 ± 1.3	50.2 ± 0.3	60.2 ± 0.3	62.1 ± 0.1	47.1 ± 0.1	25.4 ± 0.2	37.6 ± 0.1
#2704	49.8 ± 0.1	52.1 ± 1.5	57.9 ± 2.1	67.0 ± 0.7	52.4 ± 0.9	25.6 ± 0.1	38.4 ± 0.0
#2705	27.5 ± 0.2	36.4 ± 3.6	22.6 ± 3.2	36.6 ± 4.9	40.3 ± 3.9	24.1 ± 2.2	25.1 ± 2.7
#2706	31.6 ± 1.4	44.0 ± 0.1	30.0 ± 0.1	33.5 ± 2.7	50.0 ± 0.5	30.5 ± 0.6	27.2 ± 0.1
#2707	40.7 ± 0.3	53.7 ± 0.7	45.9 ± 0.1	44.4 ± 0.4	21.9 ± 1.1	18.6 ± 1.0	29.7 ± 0.8
#2708	38.1 ± 0.1	48.2 ± 1.8	35.2 ± 0.1	23.8 ± 0.6	56.6 ± 0.1	35.4 ± 0.1	26.9 ± 1.3
#2710	18.3 ± 1.4	17.4 ± 1.5	15.4 ± 1.8	18.1 ± 2.0	20.7 ± 1.5	17.4 ± 1.9	22.0 ± 1.4
#2813	40.7 ± 0.3	63.7 ± 0.3	61.6 ± 0.1	53.7 ± 0.7	21.1 ± 1.1	22.1 ± 0.7	37.3 ± 0.7
#2814	23.8 ± 0.6	44.1 ± 0.6	41.0 ± 0.1	17.8 ± 0.2	23.6 ± 0.6	27.2 ± 0.7	36.0 ± 5.0
#2815	40.4 ± 2.0	58.3 ± 2.0	38.9 ± 4.1	0.2 ± 0.1	37.2 ± 1.2	35.6 ± 0.6	5.0 ± 0.6
#2816	27.1 ± 0.7	41.8 ± 0.8	37.2 ± 0.7	34.6 ± 1.7	33.9 ± 1.3	32.4 ± 0.1	31.1 ± 0.6
#2817	23.4 ± 0.5	29.4 ± 5.3	31.5 ± 0.0	31.5 ± 3.7	14.6 ± 1.7	15.9 ± 1.5	17.1 ± 1.8
#2818	52.7 ± 0.4	44.1 ± 0.1	11.1 ± 1.6	0.7 ± 0.1	48.6 ± 0.4	20.5 ± 0.5	18.2 ± 1.9
#2819	26.6 ± 0.1	29.5 ± 0.9	34.7 ± 1.7	53.6 ± 0.0	25.6 ± 2.0	26.8 ± 0.8	33.4 ± 2.2
#2820	45.2 ± 0.3	67.2 ± 0.0	103.9 ± 6.5	4.2 ± 0.6	50.7 ± 0.3	47.3 ± 2.3	78.2 ± 4.0
#2821	31.1 ± 0.3	40.8 ± 0.0	25.8 ± 0.4	1.1 ± 0.2	32.0 ± 0.0	13.8 ± 1.6	1.2 ± 0.1
#2822	48.1 ± 1.3	27.3 ± 1.5	23.6 ± 0.8	47.5 ± 5.7	67.6 ± 0.3	25.8 ± 0.1	26.2 ± 2.7
#2823	38.1 ± 5.2	47.7 ± 0.0	40.9 ± 4.8	29.9 ± 4.2	35.9 ± 3.5	33.5 ± 3.5	41.4 ± 8.6
#2824	39.1 ± 0.2	54.7 ± 1.8	56.8 ± 0.1	47.9 ± 1.3	33.4 ± 0.7	27.6 ± 0.1	29.9 ± 0.2
#2825	18.5 ± 6.5	53.5 ± 0.2	52.8 ± 0.7	19.1 ± 1.4	35.4 ± 1.3	30.7 ± 4.6	30.8 ± 1.3
#2826	70.6 ± 0.1	67.4 ± 0.7	84.4 ± 2.4	11.5 ± 0.4	63.2 ± 0.0	13.9 ± 0.1	21.1 ± 0.0
#2832	28.1 ± 0.7	37.2 ± 0.7	32.4 ± 0.3	30.8 ± 3.0	28.3 ± 2.8	27.9 ± 0.1	14.6 ± 1.7
#2833	28.9 ± 0.1	32.9 ± 0.8	31.3 ± 1.1	23.5 ± 0.4	27.1 ± 2.2	23.9 ± 1.6	11.7 ± 0.5
#2834	34.1 ± 1.5	35.9 ± 1.4	32.1 ± 1.3	36.1 ± 2.2	35.5 ± 2.9	26.2 ± 3.0	26.7 ± 0.9
#2835	32.6 ± 1.4	42.0 ± 0.7	42.1 ± 1.2	43.6 ± 5.7	21.6 ± 1.4	17.5 ± 0.4	16.3 ± 1.7
#2836	35.7 ± 0.1	39.5 ± 1.7	33.3 ± 2.6	38.1 ± 2.6	28.4 ± 0.1	20.7 ± 0.2	26.8 ± 1.9
#2838	33.1 ± 2.0	35.7 ± 1.8	29.1 ± 1.3	38.5 ± 2.1	32.6 ± 0.1	28.5 ± 0.8	33.8 ± 1.3
#2841	30.6 ± 0.6	40.2 ± 0.1	38.2 ± 0.0	41.8 ± 0.8	24.6 ± 0.5	22.2 ± 0.4	23.9 ± 0.2
#2842	30.38 ± 0.0	35.2 ± 1.4	35.9 ± 0.4	17.5 ± 2.2	15.8 ± 1.4	15.6 ± 0.1	17.4 ± 2.0
#2850	41.4 ± 0.6	45.1 ± 0.5	40.8 ± 5.4	29.3 ± 3.2	37.4 ± 0.9	26.3 ± 0.2	19.3 ± 1.9
#3600	27.7 ± 0.2	24.4 ± 3.3	17.5 ± 2.3	25.6 ± 2.6	31.9 ± 3.4	24.2 ± 2.8	24.1 ± 2.4

Table S3 Maximum growth rates (μ_{max} [h^{-1}]) of all 72 strains obtained during biodiversity screening. Whole set of growth screening results using the Growth Profiler device by Enzyscreen with different acetate and formate concentrations as co-substrates in combination with 20 g L^{-1} glucose (n=2).

Strain	Glucose	2.5 g L^{-1} Acetate	5 g L^{-1} Acetate	10 g L^{-1} Acetate	2.5 g L^{-1} Formate	5 g L^{-1} Formate	10 g L-1 Formate
#1946	0.33 ± 0.01	0.29 ± 0.02	0.26 ± 0.03	0.25 ± 0.01	0.29 ± 0.00	0.32 ± 0.01	0.30 ± 0.02
#1947	0.26 ± 0.02	0.30 ± 0.00	0.25 ± 0.00	0.23 ± 0.01	0.28 ± 0.00	0.29 ± 0.00	0.41 ± 0.00
#1949	0.17 ± 0.03	0.12 ± 0.00	0.12 ± 0.00	0.11 ± 0.00	0.09 ± 0.01	0.10 ± 0.02	0.04 ± 0.00
#1951	0.30 ± 0.01	0.23 ± 0.01	0.25 ± 0.00	0.21 ± 0.02	0.27 ± 0.00	0.25 ± 0.01	0.22 ± 0.02
#2134	0.32 ± 0.04	0.26 ± 0.00	0.22 ± 0.01	0.20 ± 0.01	0.31 ± 0.00	0.23 ± 0.02	0.22 ± 0.01
#2135	0.30 ± 0.02	0.29 ± 0.02	0.29 ± 0.00	0.25 ± 0.00	0.31 ± 0.01	0.27 ± 0.01	0.19 ± 0.00
#2136	0.40 ± 0.00	0.33 ± 0.02	0.25 ± 0.00	0.23 ± 0.00	0.26 ± 0.01	0.27 ± 0.00	0.18 ± 0.00
#2144	0.28 ± 0.00	0.29 ± 0.00	0.26 ± 0.00	0.20 ± 0.00	0.30 ± 0.00	0.23 ± 0.01	0.29 ± 0.00
#2152	0.29 ± 0.01	0.30 ± 0.01	0.26 ± 0.02	0.17 ± 0.00	0.31 ± 0.00	0.25 ± 0.01	0.20 ± 0.00
#2158	0.26 ± 0.01	0.29 ± 0.00	0.24 ± 0.01	0.25 ± 0.01	0.27 ± 0.01	0.22 ± 0.00	0.22 ± 0.00
#2162	0.26 ± 0.05	0.25 ± 0.00	0.24 ± 0.01	0.20 ± 0.00	0.21 ± 0.01	0.21 ± 0.03	0.17 ± 0.02
#2167	0.34 ± 0.02	0.31 ± 0.00	0.28 ± 0.00	0.25 ± 0.01	0.27 ± 0.01	0.26 ± 0.00	0.21 ± 0.01
#2169	0.25 ± 0.02	0.28 ± 0.00	0.26 ± 0.01	0.19 ± 0.02	0.27 ± 0.00	0.31 ± 0.01	0.20 ± 0.00
#2172	0.22 ± 0.00	0.29 ± 0.00	0.28 ± 0.00	0.21 ± 0.00	0.22 ± 0.00	0.19 ± 0.00	0.19 ± 0.00
#2177	0.25 ± 0.01	0.32 ± 0.00	0.26 ± 0.01	0.20 ± 0.00	0.27 ± 0.02	0.24 ± 0.00	0.18 ± 0.00
#2178	0.37 ± 0.02	0.24 ± 0.03	0.21 ± 0.01	0.00 ± 0.00	0.27 ± 0.01	0.28 ± 0.00	0.05 ± 0.00
#2179	0.22 ± 0.01	0.29 ± 0.01	0.27 ± 0.01	0.17 ± 0.01	0.24 ± 0.03	0.23 ± 0.00	0.22 ± 0.00
#2196	0.33 ± 0.02	0.28 ± 0.01	0.29 ± 0.00	0.22 ± 0.02	0.26 ± 0.00	0.29 ± 0.00	0.22 ± 0.00
#2197	0.28 ± 0.02	0.27 ± 0.00	0.29 ± 0.01	0.15 ± 0.01	0.21 ± 0.00	0.30 ± 0.02	0.20 ± 0.01
#2199	0.25 ± 0.00	0.28 ± 0.00	0.25 ± 0.01	0.21 ± 0.01	0.28 ± 0.01	0.20 ± 0.01	0.25 ± 0.00
#2205	0.15 ± 0.00	0.21 ± 0.02	0.19 ± 0.01	0.07 ± 0.00	0.17 ± 0.00	0.00 ± 0.00	0.14 ± 0.01
#2208	0.21 ± 0.01	0.20 ± 0.00	0.19 ± 0.00	0.15 ± 0.03	0.16 ± 0.01	0.20 ± 0.00	0.17 ± 0.02
#2209	0.22 ± 0.00	0.24 ± 0.01	0.20 ± 0.00	0.14 ± 0.02	0.23 ± 0.00	0.19 ± 0.01	0.18 ± 0.00
#2210	0.16 ± 0.00	0.14 ± 0.01	0.19 ± 0.00	0.00 ± 0.00	0.06 ± 0.01	0.14 ± 0.00	0.14 ± 0.00
#2211	0.24 ± 0.01	0.14 ± 0.01	0.14 ± 0.02	0.24 ± 0.01	0.16 ± 0.02	0.20 ± 0.00	0.20 ± 0.0
#2212	0.26 ± 0.04	0.19 ± 0.01	0.23 ± 0.00	0.12 ± 0.00	0.13 ± 0.00	0.12 ± 0.02	0.20 ± 0.02
#2213	0.20 ± 0.00	0.21 ± 0.02	0.18 ± 0.00	0.00 ± 0.00	0.17 ± 0.01	0.18 ± 0.00	0.20 ± 0.01
#2214	0.25 ± 0.02	0.25 ± 0.00	0.17 ± 0.00	0.12 ± 0.01	0.25 ± 0.01	0.19 ± 0.01	0.11 ± 0.01
#2215	0.29 ± 0.01	0.24 ± 0.00	0.20 ± 0.02	0.10 ± 0.02	0.24 ± 0.00	0.21 ± 0.00	0.15 ± 0.01
#2218	0.29 ± 0.02	0.32 ± 0.00	0.27 ± 0.01	0.00 ± 0.00	0.30 ± 0.01	0.30 ± 0.00	0.08 ± 0.00
#2219	0.18 ± 0.00	0.29 ± 0.00	0.20 ± 0.03	0.19 ± 0.00	0.21 ± 0.01	0.15 ± 0.01	0.23 ± 0.00
#2220	0.25 ± 0.01	0.25 ± 0.01	0.16 ± 0.00	0.21 ± 0.01	0.20 ± 0.01	0.19 ± 0.00	0.01 ± 0.01
#2221	0.28 ± 0.01	0.37 ± 0.00	0.25 ± 0.00	0.13 ± 0.00	0.28 ± 0.01	0.25 ± 0.00	0.12 ± 0.00
#2229	0.24 ± 0.01	0.32 ± 0.00	0.21 ± 0.02	0.18 ± 0.00	0.24 ± 0.00	0.24 ± 0.00	0.20 ± 0.02
#2696	0.30 ± 0.01	0.26 ± 0.00	0.19 ± 0.01	0.25 ± 0.03	0.26 ± 0.00	0.24 ± 0.00	0.21 ± 0.01
#2697	0.26 ± 0.01	0.21 ± 0.00	0.29 ± 0.00	0.17 ± 0.01	0.17 ± 0.01	0.23 ± 0.00	0.22 ± 0.00
#2698	0.26 ± 0.01	0.28 ± 0.00	0.27 ± 0.01	0.18 ± 0.01	0.26 ± 0.03	0.24 ± 0.04	0.21 ± 0.02
#2699	0.23 ± 0.01	0.31 ± 0.01	0.29 ± 0.00	0.22 ± 0.02	0.25 ± 0.00	0.25 ± 0.00	0.25 ± 0.00
#2700	0.27 ± 0.00	0.31 ± 0.01	0.26 ± 0.03	0.20 ± 0.01	0.26 ± 0.01	0.27 ± 0.00	0.22 ± 0.00
#2701	0.33 ± 0.03	0.33 ± 0.02	0.33 ± 0.01	0.24 ± 0.01	0.28 ± 0.01	0.29 ± 0.01	0.25 ± 0.00
#2702	0.28 ± 0.01	0.34 ± 0.01	0.31 ± 0.01	0.21 ± 0.01	0.27 ± 0.01	0.29 ± 0.00	0.25 ± 0.00
#2703	0.36 ± 0.01	0.37 ± 0.00	0.34 ± 0.00	0.27 ± 0.00	0.32 ± 0.00	0.27 ± 0.00	0.28 ± 0.00
#2704	0.32 ± 0.01	0.32 ± 0.00	0.28 ± 0.00	0.24 ± 0.00	0.29 ± 0.00	0.27 ± 0.00	0.29 ± 0.01
#2705	0.19 ± 0.03	0.23 ± 0.02	0.18 ± 0.04	0.12 ± 0.00	0.18 ± 0.00	0.19 ± 0.01	0.14 ± 0.00
#2706	0.19 ± 0.00	0.17 ± 0.01	0.17 ± 0.01	0.10 ± 0.00	0.12 ± 0.00	0.17 ± 0.00	0.15 ± 0.00
#2707	0.24 ± 0.01	0.25 ± 0.00	0.19 ± 0.00	0.19 ± 0.00	0.18 ± 0.00	0.18 ± 0.00	0.18 ± 0.00
#2708	0.15 ± 0.01	0.20 ± 0.00	0.15 ± 0.01	0.09 ± 0.00	0.14 ± 0.01	0.17 ± 0.00	0.14 ± 0.00
#2710	0.18 ± 0.02	0.24 ± 0.00	0.22 ± 0.01	0.11 ± 0.00	0.18 ± 0.01	0.22 ± 0.00	0.17 ± 0.02
#2813	0.20 ± 0.01	0.21 ± 0.02	0.20 ± 0.01	0.16 ± 0.00	0.17 ± 0.02	0.16 ± 0.02	0.19 ± 0.01
#2814	0.35 ± 0.00	0.19 ± 0.00	0.19 ± 0.00	0.17 ± 0.00	0.27 ± 0.00	0.22 ± 0.02	0.21 ± 0.01
#2815	0.28 ± 0.00	0.16 ± 0.01	0.08 ± 0.01	0.02 ± 0.02	0.14 ± 0.01	0.13 ± 0.01	0.08 ± 0.00
#2816	0.30 ± 0.01	0.17 ± 0.01	0.15 ± 0.00	0.18 ± 0.00	0.24 ± 0.02	0.22 ± 0.02	0.22 ± 0.01
#2817	0.26 ± 0.00	0.18 ± 0.00	0.19 ± 0.02	0.19 ± 0.01	0.20 ± 0.02	0.15 ± 0.02	0.12 ± 0.01
#2818	0.12 ± 0.00	0.13 ± 0.00	0.10 ± 0.00	0.00 ± 0.00	0.16 ± 0.00	0.06 ± 0.00	0.08 ± 0.00
#2819	0.34 ± 0.03	0.28 ± 0.03	0.23 ± 0.01	0.25 ± 0.01	0.22 ± 0.00	0.24 ± 0.01	0.22 ± 0.01
#2820	0.32 ± 0.03	0.16 ± 0.02	0.29 ± 0.02	0.60 ± 0.02	0.18 ± 0.00	0.17 ± 0.02	0.46 ± 0.00
#2821	0.26 ± 0.00	0.25 ± 0.00	0.18 ± 0.01	0.00 ± 0.00	0.22 ± 0.00	0.21 ± 0.00	0.00 ± 0.00
#2822	0.32 ± 0.00	0.24 ± 0.00	0.23 ± 0.01	0.25 ± 0.03	0.37 ± 0.01	0.24 ± 0.02	0.26 ± 0.00
#2823	0.53 ± 0.04	0.27 ± 0.01	0.27 ± 0.04	0.37 ± 0.03	0.33 ± 0.01	0.31 ± 0.01	0.25 ± 0.06
#2824	0.22 ± 0.00	0.20 ± 0.00	0.19 ± 0.00	0.20 ± 0.00	0.16 ± 0.01	0.18 ± 0.00	0.17 ± 0.00
#2825	0.22 ± 0.02	0.18 ± 0.00	0.17 ± 0.03	0.27 ± 0.01	0.17 ± 0.01	0.08 ± 0.01	0.23 ± 0.02
#2826	0.24 ± 0.00	0.23 ± 0.00	0.22 ± 0.01	0.12 ± 0.00	0.24 ± 0.00	0.00 ± 0.00	0.13 ± 0.00
#2832	0.31 ± 0.00	0.22 ± 0.00	0.20 ± 0.01	0.21 ± 0.00	0.23 ± 0.00	0.21 ± 0.00	0.21 ± 0.03
#2833	0.34 ± 0.02	0.26 ± 0.01	0.20 ± 0.00	0.19 ± 0.03	0.21 ± 0.01	0.22 ± 0.01	0.11 ± 0.01
#2834	0.25 ± 0.07	0.24 ± 0.00	0.20 ± 0.00	0.23 ± 0.01	0.28 ± 0.01	0.23 ± 0.03	0.19 ± 0.00
#2835	0.37 ± 0.02	0.27 ± 0.02	0.24 ± 0.00	0.21 ± 0.00	0.27 ± 0.00	0.25 ± 0.00	0.18 ± 0.00
#2836	0.35 ± 0.02	0.25 ± 0.02	0.19 ± 0.01	0.26 ± 0.01	0.29 ± 0.07	0.41 ± 0.02	0.18 ± 0.01
#2838	0.32 ± 0.00	0.23 ± 0.00	0.19 ± 0.01	0.28 ± 0.08	0.21 ± 0.00	0.22 ± 0.00	0.24 ± 0.00
#2841	0.39 ± 0.11	0.22 ± 0.00	0.21 ± 0.00	0.30 ± 0.01	0.37 ± 0.05	0.33 ± 0.00	0.22 ± 0.00
#2842	0.42 ± 0.01	0.33 ± 0.02	0.26 ± 0.00	0.26 ± 0.01	0.23 ± 0.00	0.31 ± 0.00	0.21 ± 0.03
#2850	0.28 ± 0.01	0.16 ± 0.00	0.17 ± 0.00	0.24 ± 0.00	0.14 ± 0.00	0.10 ± 0.00	0.17 ± 0.00
#3600	0.23 ± 0.00	0.27 ± 0.01	0.25 ± 0.00	0.15 ± 0.00	0.22 ± 0.01	0.30 ± 0.03	0.16 ± 0.05

Table S4 pH [-] values of all 72 strains obtained during biodiversity screening. Whole set of growth screening results using the Growth Profiler device by Enzyscreen with different acetate and formate concentrations as co-substrates in combination with 20 g L^{-1} glucose (n=2).

Strain	Glucose	2.5 g L^{-1} Acetate	5 g L^{-1} Acetate	10 g L^{-1} Acetate	2.5 g L^{-1} Formate	5 g L^{-1} Formate	10 g L-1 Formate
#1946	5.51 ± 0.02	6.20 ± 0.00	6.70 ± 0.01	8.84 ± 0.05	6.42 ± 0.00	7.46 ± 0.00	8.89 ± 0.00
#1947	5.64 ± 0.00	6.22 ± 0.00	6.77 ± 0.01	8.89 ± 0.03	6.42 ± 0.00	7.50 ± 0.00	8.95 ± 0.00
#1949	5.37 ± 0.01	5.90 ± 0.00	6.46 ± 0.01	8.22 ± 0.01	6.40 ± 0.02	6.96 ± 0.00	7.99 ± 0.01
#1951	5.70 ± 0.00	6.19 ± 0.01	6.35 ± 0.01	8.51 ± 0.00	6.74 ± 0.00	7.80 ± 0.30	7.52 ± 0.00
#2134	5.92 ± 0.01	6.56 ± 0.02	7.36 ± 0.01	9.03 ± 0.03	6.87 ± 0.00	7.75 ± 0.01	7.39 ± 0.00
#2135	5.78 ± 0.02	6.33 ± 0.00	6.89 ± 0.03	8.60 ± 0.00	6.88 ± 0.00	7.37 ± 0.00	7.91 ± 0.00
#2136	5.85 ± 0.01	6.35 ± 0.00	6.83 ± 0.00	8.62 ± 0.02	6.89 ± 0.00	7.54 ± 0.01	7.86 ± 0.02
#2144	5.87 ± 0.01	6.43 ± 0.00	7.30 ± 0.02	8.97 ± 0.00	6.78 ± 0.01	8.01 ± 0.01	8.28 ± 0.00
#2152	5.89 ± 0.00	6.43 ± 0.00	6.98 ± 0.01	8.43 ± 0.06	6.86 ± 0.01	7.82 ± 0.01	8.49 ± 0.01
#2158	5.85 ± 0.01	6.40 ± 0.00	7.30 ± 0.16	9.08 ± 0.01	6.69 ± 0.01	7.96 ± 0.00	8.31 ± 0.00
#2162	5.64 ± 0.00	6.16 ± 0.00	6.68 ± 0.00	8.82 ± 0.03	6.25 ± 0.00	6.26 ± 0.00	6.95 ± 0.01
#2167	5.56 ± 0.00	6.10 ± 0.00	6.59 ± 0.01	8.90 ± 0.01	6.55 ± 0.00	6.41 ± 0.00	7.66 ± 0.06
#2169	5.46 ± 0.00	6.05 ± 0.00	6.54 ± 0.00	8.80 ± 0.07	6.36 ± 0.01	6.41 ± 0.00	8.04 ± 0.00
#2172	5.52 ± 0.00	6.09 ± 0.00	6.57 ± 0.00	8.87 ± 0.00	6.39 ± 0.00	6.54 ± 0.00	7.72 ± 0.01
#2177	5.68 ± 0.00	6.22 ± 0.00	6.73 ± 0.01	8.38 ± 0.00	6.74 ± 0.00	7.72 ± 0.01	7.70 ± 0.02
#2178	5.72 ± 0.00	6.17 ± 0.00	6.80 ± 0.00	6.61 ± 0.01	6.60 ± 0.03	8.20 ± 0.01	7.11 ± 0.05
#2179	5.63 ± 0.00	6.24 ± 0.00	6.74 ± 0.00	8.33 ± 0.00	6.79 ± 0.00	7.00 ± 0.00	7.30 ± 0.03
#2196	5.55 ± 0.00	6.07 ± 0.00	6.57 ± 0.01	8.69 ± 0.00	6.53 ± 0.00	6.43 ± 0.01	7.54 ± 0.02
#2197	5.66 ± 0.00	6.18 ± 0.00	6.73 ± 0.02	8.95 ± 0.03	5.98 ± 0.02	6.23 ± 0.07	7.76 ± 0.00
#2199	5.73 ± 0.01	6.30 ± 0.00	6.95 ± 0.04	8.88 ± 0.00	6.73 ± 0.01	7.97 ± 0.02	8.31 ± 0.00
#2205	5.63 ± 0.00	6.17 ± 0.00	6.65 ± 0.00	7.57 ± 0.06	6.44 ± 0.00	6.97 ± 0.01	8.09 ± 0.09
#2208	5.58 ± 0.00	6.15 ± 0.00	6.62 ± 0.01	7.58 ± 0.02	6.44 ± 0.00	6.44 ± 0.00	7.83 ± 0.02
#2209	5.66 ± 0.01	6.23 ± 0.01	6.92 ± 0.03	7.89 ± 0.05	6.69 ± 0.01	7.89 ± 0.00	7.94 ± 0.01
#2210	5.52 ± 0.00	6.50 ± 0.01	6.92 ± 0.01	8.04 ± 0.02	5.84 ± 0.00	5.97 ± 0.00	8.07 ± 0.05
#2211	5.86 ± 0.01	6.26 ± 0.00	6.44 ± 0.01	8.70 ± 0.03	6.48 ± 0.10	6.71 ± 0.01	8.64 ± 0.01
#2212	5.64 ± 0.00	6.21 ± 0.00	6.58 ± 0.04	8.73 ± 0.04	6.06 ± 0.00	6.25 ± 0.00	6.54 ± 0.01
#2213	5.64 ± 0.00	6.19 ± 0.01	7.28 ± 0.00	6.46 ± 0.00	6.50 ± 0.00	7.52 ± 0.17	6.65 ± 0.01
#2214	6.00 ± 0.00	6.48 ± 0.00	7.30 ± 0.04	8.19 ± 0.05	6.27 ± 0.00	6.29 ± 0.00	7.84 ± 0.04
#2215	5.68 ± 0.00	6.21 ± 0.00	7.03 ± 0.03	8.67 ± 0.03	6.00 ± 0.00	6.02 ± 0.00	8.67 ± 0.06
#2218	5.72 ± 0.00	6.20 ± 0.00	7.65 ± 0.03	6.46 ± 0.00	6.41 ± 0.00	7.69 ± 0.01	6.92 ± 0.01
#2219	5.72 ± 0.00	6.22 ± 0.00	7.65 ± 0.03	8.77 ± 0.00	6.37 ± 0.00	8.04 ± 0.01	8.31 ± 0.03
#2220	5.66 ± 0.00	6.20 ± 0.00	7.61 ± 0.01	8.61 ± 0.04	6.35 ± 0.01	6.38 ± 0.01	6.52 ± 0.02
#2221	5.81 ± 0.00	6.32 ± 0.00	7.67 ± 0.00	8.15 ± 0.00	6.62 ± 0.00	6.49 ± 0.00	6.16 ± 0.00
#2229	5.80 ± 0.00	6.27 ± 0.00	7.71 ± 0.00	8.88 ± 0.02	6.63 ± 0.00	6.47 ± 0.00	7.71 ± 0.89
#2696	5.77 ± 0.00	6.34 ± 0.00	7.71 ± 0.04	8.74 ± 0.02	6.51 ± 0.00	7.77 ± 0.00	8.98 ± 0.00
#2697	5.81 ± 0.00	6.32 ± 0.01	6.99 ± 0.02	8.50 ± 0.00	6.52 ± 0.02	7.82 ± 0.03	8.93 ± 0.00
#2698	5.53 ± 0.00	6.11 ± 0.01	7.68 ± 0.03	8.73 ± 0.01	6.27 ± 0.00	7.93 ± 0.02	8.71 ± 0.02
#2699	5.61 ± 0.01	6.22 ± 0.00	6.78 ± 0.01	8.47 ± 0.01	8.37 ± 0.00	7.81 ± 0.00	8.35 ± 0.02
#2700	5.38 ± 0.30	6.28 ± 0.01	6.96 ± 0.01	8.31 ± 0.00	6.69 ± 0.00	7.78 ± 0.02	8.21 ± 0.01
#2701	5.73 ± 0.01	6.34 ± 0.01	7.01 ± 0.02	8.27 ± 0.01	6.70 ± 0.01	7.82 ± 0.01	8.31 ± 0.02
#2702	6.03 ± 0.01	6.48 ± 0.00	7.20 ± 0.00	8.33 ± 0.00	6.77 ± 0.00	7.84 ± 0.01	8.06 ± 0.01
#2703	5.68 ± 0.02	6.37 ± 0.00	7.25 ± 0.01	8.76 ± 0.00	6.62 ± 0.00	7.70 ± 0.05	8.61 ± 0.01
#2704	5.71 ± 0.00	6.35 ± 0.00	7.24 ± 0.00	8.78 ± 0.00	6.55 ± 0.00	7.93 ± 0.20	8.55 ± 0.01
#2705	5.65 ± 0.01	6.51 ± 0.00	7.11 ± 0.02	8.14 ± 0.00	6.82 ± 0.02	7.16 ± 0.01	7.14 ± 0.00
#2706	5.90 ± 0.02	6.51 ± 0.00	7.12 ± 0.04	8.25 ± 0.07	6.80 ± 0.01	7.67 ± 0.00	6.56 ± 0.04
#2707	6.06 ± 0.04	6.49 ± 0.06	7.20 ± 0.10	8.55 ± 0.01	6.73 ± 0.01	6.68 ± 0.03	6.18 ± 0.00
#2708	5.97 ± 0.02	6.55 ± 0.00	7.24 ± 0.02	8.11 ± 0.00	6.81 ± 0.00	7.74 ± 0.13	6.64 ± 0.01
#2710	6.23 ± 0.00	6.69 ± 0.00	7.33 ± 0.00	8.41 ± 0.03	6.91 ± 0.00	8.08 ± 0.00	8.07 ± 0.04
#2813	5.98 ± 0.02	6.46 ± 0.02	7.21 ± 0.00	8.25 ± 0.01	6.62 ± 0.00	6.17 ± 0.00	7.88 ± 0.04
#2814	5.81 ± 0.01	6.19 ± 0.01	6.85 ± 0.02	8.14 ± 0.00	6.27 ± 0.00	7.90 ± 0.00	8.51 ± 0.01
#2815	5.70 ± 0.07	6.23 ± 0.01	6.85 ± 0.02	6.48 ± 0.00	6.56 ± 0.02	7.76 ± 0.00	8.48 ± 0.02
#2816	5.97 ± 0.00	6.23 ± 0.01	6.96 ± 0.00	8.61 ± 0.01	6.44 ± 0.00	7.98 ± 0.00	8.22 ± 0.01
#2817	5.78 ± 0.01	6.43 ± 0.00	6.82 ± 0.00	8.51 ± 0.00	6.20 ± 0.00	6.21 ± 0.02	6.39 ± 0.02
#2818	5.76 ± 0.01	6.22 ± 0.02	6.40 ± 0.01	6.50 ± 0.01	6.43 ± 0.01	6.42 ± 0.09	6.46 ± 0.06
#2819	6.04 ± 0.00	6.25 ± 0.00	7.16 ± 0.02	8.23 ± 0.01	6.57 ± 0.00	7.85 ± 0.00	8.43 ± 0.02
#2820	5.95 ± 0.00	6.56 ± 0.00	7.11 ± 0.01	7.96 ± 0.01	6.57 ± 0.00	7.98 ± 0.01	8.52 ± 0.00
#2821	5.78 ± 0.01	6.33 ± 0.00	7.67 ± 0.00	6.50 ± 0.04	6.62 ± 0.00	7.98 ± 0.01	7.93 ± 0.01
#2822	4.18 ± 0.03	6.46 ± 0.00	4.89 ± 0.02	8.92 ± 0.04	6.19 ± 0.01	7.97 ± 0.06	8.54 ± 0.02
#2823	5.79 ± 0.05	4.34 ± 0.01	6.85 ± 0.00	8.28 ± 0.01	6.49 ± 0.00	7.83 ± 0.00	8.78 ± 0.00
#2824	6.01 ± 0.01	6.29 ± 0.00	7.16 ± 0.02	8.35 ± 0.01	6.82 ± 0.00	7.85 ± 0.01	6.92 ± 0.00
#2825	5.82 ± 0.08	6.45 ± 0.01	6.94 ± 0.01	8.08 ± 0.01	6.72 ± 0.00	7.65 ± 0.03	8.12 ± 0.00
#2826	5.66 ± 0.01	6.38 ± 0.15	6.86 ± 0.01	8.24 ± 0.04	6.38 ± 0.01	7.13 ± 0.63	6.21 ± 0.01
#2832	5.85 ± 0.01	6.19 ± 0.02	7.09 ± 0.01	8.71 ± 0.01	6.56 ± 0.00	7.74 ± 0.01	8.22 ± 0.00
#2833	5.60 ± 0.01	6.47 ± 0.00	6.81 ± 0.02	6.96 ± 0.01	6.45 ± 0.00	7.85 ± 0.02	7.91 ± 0.04
#2834	5.78 ± 0.00	6.17 ± 0.01	7.09 ± 0.01	8.52 ± 0.03	6.59 ± 0.02	7.91 ± 0.01	8.60 ± 0.03
#2835	5.75 ± 0.00	6.31 ± 0.00	7.04 ± 0.01	8.56 ± 0.00	6.65 ± 0.00	8.03 ± 0.01	8.49 ± 0.00
#2836	5.69 ± 0.01	6.52 ± 0.00	7.31 ± 0.00	8.60 ± 0.00	6.58 ± 0.00	7.94 ± 0.02	8.76 ± 0.02
#2838	5.59 ± 0.00	6.23 ± 0.00	7.06 ± 0.00	8.63 ± 0.01	6.48 ± 0.00	7.76 ± 0.00	8.74 ± 0.00
#2841	5.63 ± 0.00	6.22 ± 0.00	6.87 ± 0.00	8.55 ± 0.01	6.56 ± 0.00	7.79 ± 0.00	8.70 ± 0.02
#2842	5.77 ± 0.00	6.27 ± 0.00	6.97 ± 0.00	8.59 ± 0.02	6.82 ± 0.00	8.09 ± 0.00	8.44 ± 0.02
#2850	5.64 ± 0.06	6.21 ± 0.00	6.56 ± 0.02	8.61 ± 0.03	6.32 ± 0.03	7.06 ± 0.08	8.35 ± 0.02
#3600	5.77 ± 0.00	6.28 ± 0.00	6.75 ± 0.00	8.54 ± 0.02	6.66 ± 0.00	6.90 ± 0.01	6.72 ± 0.01

Table S5 Production parameters of biodiversity screening for acetate co-utilization. Growth results of the five most promising strains obtained during experiments using the Growth Profiler device by Enzyscreen with different acetate concentrations as co-substrates in combination with 20 g L^{-1} glucose (n=2). *U. maydis* #2135 and #2136 obtained best results using 10 g L^{-1} acetate. Remaining strains showed highest growth using 2.5 g L^{-1} acetate.

Strain	C-source	Max. OD_{600nm} [-]	$Y_{OD/S}$ [max. OD_{600nm}/$g_{c\text{-}source}$]	$Y_{OD/S}$ [max. OD_{600nm}/C-$mol_{c\text{-}source}$]
U. maydis #1946	Glucose	46.8	2.34	69.9
		48.3	2.42	72.1
	Glucose + Acetate	55.9	2.48	77.6
		56.5	2.51	78.5
U. cynodontis #2707	Glucose	40.7	2.03	60.7
		41.3	2.07	61.7
	Glucose + Acetate	53.7	2.38	74.5
		54.0	2.40	75.0
U. maydis #2136	Glucose	29.9	1.40	44.6
		19.9	1.00	29.8
	Glucose + Acetate*	51.4	1.71	56.5
		52.8	1.76	58.1
U. maydis #2135	Glucose	19.7	0.99	29.4
		22.5	1.13	33.6
	Glucose + Acetate*	49.5	1.65	54.4
		49.8	1.66	54.8
U. rabenhorstiana #2708	Glucose	38.0	1.90	56.8
		38.2	1.91	57.0
	Glucose + Acetate	48.3	2.14	67.0
		50.0	2.22	69.5

Table S6 Production parameters of biodiversity screening for formate co-utilization. Growth results of the five most promising strains obtained during experiments using the Growth Profiler device by Enzyscreen with 2.5 g L^{-1} formate as co-substrates in combination with 20 g L^{-1} glucose (n=2).

Strain	C-source	Max. OD_{600nm} [-]	$Y_{OD/S}$ [max. OD_{600nm}/$g_{c\text{-}source}$]	$Y_{OD/S}$ [max. OD_{600nm}/C-$mol_{c\text{-}source}$]
U. rabenhorstiana #2708	Glucose	38.0	1.90	56.8
		38.2	1.91	57.0
	Glucose + Formate	56.6	2.52	80.9
		56.7	2.52	81.0
U. cynodontis #2707	Glucose	31.6	1.58	47.2
		33.0	1.65	49.3
	Glucose + Formate	50.0	2.22	71.5
		50.5	2.25	72.2
U. maydis #2177	Glucose	29.0	1.45	43.3
		30.2	1.51	45.0
	Glucose + Formate	42.0	1.86	59.9
		43.1	1.91	61.5
U. cynodontis #2706	Glucose	27.5	1.37	41.0
		27.7	1.38	41.3
	Glucose + Formate	40.4	1.70	57.6
		44.2	1.93	63.1
U. maydis #2196	Glucose	36.3	1.82	54.2
		37.8	1.89	56.4
	Glucose + Formate	42.3	1.88	60.4
		43.4	1.93	62.0

Table S7 Production parameters of System Duetz cultivation for acetate co-utilization. System Duetz® 24-deep well plates cultivation experiments were performed with 1.5 ml MTM medium and 50 g L^{-1} glucose. 6.25 g L^{-1} acetate were added during co-culture cultivations. Error bars indicate the deviation from the mean for n=2.

Strain	C-source	$Titer_{max}$ [g L^{-1}]	$Y_{P/S}$ [$g_{ITA}/g_{c\text{-}source}$]	$Y_{P/S}$ [$C\text{-}mol_{ITA}/C\text{-}mol_{c\text{-}source}$]	Max. OD [-]
U. maydis #2229	Glucose	2.99	0.060	0.069	39
		3.38	0.068	0.078	33
	Glucose + Acetate	7.20	0.144	0.152	49
		7.60	0.152	0.161	48
U. rabenhorstiana #2708	Glucose	4.30	0.086	0.099	39
		4.31	0.101	0.099	40
	Glucose + Acetate	6.88	0.136	0.144	41
		6.80	0.138	0.145	45
U. cynodontis #2707	Glucose	2.24	0.045	0.052	53
		2.05	0.041	0.047	52
	Glucose + Acetate	2.88	0.056	0.060	50
		2.88	0.058	0.061	53
U. maydis #2135	Glucose	2.40	0.048	0.055	46
		2.42	0.048	0.056	47
	Glucose + Acetate	2.97	0.071	0.075	54
		2.35	0.059	0.063	58
U. maydis #2136	Glucose	2.36	0.047	0.054	50
		2.28	0.046	0.053	51
	Glucose + Acetate	2.32	0.046	0.049	49
		2.22	0.044	0.047	50
U. maydis #1946	Glucose	0.09	0.002	0.002	71
		0.06	0.001	0.001	73
	Glucose + Acetate	0.01	0.000	0.00	72
		0.01	0.000	0.00	66

Table S8 Production parameters of System Duetz cultivation for formate co-utilization. System Duetz® 24-deep well plates cultivation experiments were performed with 1.5 ml MTM medium and 50 g L^{-1} glucose. 6.25 g L^{-1} formate were added during co-culture cultivations. Error bars indicate the deviation from the mean for n=2.

Strain	C-source	$Titer_{max.}$ [g L^{-1}]	$Y_{P/S}$ [$g_{ITA}/g_{c\text{-}source}$]	$Y_{P/S}$ [$C\text{-}mol_{ITA}/C\text{-}mol_{c\text{-}source}$]	Max. OD [-]
U. cynodontis #2706	Glucose	6.64	0.133	0.153	51
		6.90	0.138	0.159	50
	Glucose + Formate	6.90	0.123	0.151	38
		5.81	0.103	0.127	36
U. cynodontis #2705	Glucose	6.18	0.124	0.143	52
		6.32	0.126	0.146	57
	Glucose + Formate	9.04	0.161	0.198	50
		8.20	0.146	0.179	54
U. rabenhorstiana #2708	Glucose	4.16	0.083	0.096	48
		4.35	0.087	0.100	50
	Glucose + Formate	3,98	0.071	0.087	35
		2.64	0.047	0.058	37
U. maydis #2229	Glucose	2.61	0.052	0.060	50
		2,87	0.057	0.066	54
	Glucose + Formate	0.30	0.005	0.007	37
		0.31	0.004	0.007	36
U. maydis #2177	Glucose	2.78	0.056	0.064	66
		2.60	0.052	0.060	65
	Glucose + Formate	0.43	0.008	0.009	65
		0.49	0.009	0.011	66
U. maydis #2196	Glucose	0.66	0.013	0.015	79
		0.55	0.011	0.013	80
	Glucose + Formate	0.04	0.001	0.001	60
		0.04	0.001	0.001	57

Table S9 pH values during System Duetz cultivation for acetate co-utilization. System Duetz® 24-deep well plates cultivation experiments were performed with 1.5 ml MTM medium and 50 g L^{-1} glucose. 6.25 g L^{-1} acetate were added during co-culture cultivations. Error bars indicate the deviation from the mean for n=2.

Strain	C-source	Min. pH [-]	pH End [-]
U. maydis #2229	Glucose	4.98	5.76
		4.96	5.80
	Glucose + Acetate	5.49	5.78
		5.48	5.80
U. rabenhorstiana #2708	Glucose	2.51	2.86
		2.47	2.85
	Glucose + Acetate	3.11	3.36
		3.13	3.37
U. cynodontis #2707	Glucose	3.37	3.44
		3.44	3.45
	Glucose + Acetate	3.79	3.79
		3.78	3.78
U. maydis #2135	Glucose	4.64	5.68
		4.70	5.71
	Glucose + Acetate	5.07	5.85
		5.09	5.80
U. maydis #2136	Glucose	5.02	5.72
		5.03	5.74
	Glucose + Acetate	5.36	5.78
		5.38	5.79
U. maydis #1946	Glucose	5.61	5.63
		5.64	5.70
	Glucose + Acetate	6.34	6.43
		6.32	6.42

Table S10 pH values during System Duetz cultivation for formate co-utilization. System Duetz® 24-deep well plates cultivation experiments were performed with 1.5 ml MTM medium and 50 g L^{-1} glucose. 6.25 g L^{-1} formate were added during co-culture cultivations. Error bars indicate the deviation from the mean for n=2.

Strain	C-source	Min. pH [-]	pH End [-]
U. cynodontis #2706	Glucose	3.04	3.07
		3.03	3.01
	Glucose + Formate	4.96	4.96
		4.94	4.94
U. cynodontis #2705	Glucose	2.86	2.86
		2.83	2.83
	Glucose + Formate	4.26	4.26
		4.34	4.34
U. rabenhorstiana #2708	Glucose	3.80	3.95
		3.76	3.84
	Glucose + Formate	5.08	5.08
		5.11	5.11
U. maydis #2229	Glucose	4.85	5.87
		4.87	5.88
	Glucose + Formate	5.54	6.05
		5.56	6.06
U. maydis #2177	Glucose	5.11	5.60
		5.10	5.56
	Glucose + Formate	5.64	6.57
		5.65	6.56
U. maydis #2196	Glucose	6.71	7.80
		6.75	7.09
	Glucose + Formate	6.66	7.69
		6.70	7.79

Table S11 Welch t-test results obtained for *U. maydis* #2229, *U. rabenhorstiana* #2708, and *U. cynodontis* #2705 during small-scale production and bioreactor experiments.

t-Test: Two-Sample Assuming Unequal Variances		
#2229 Maximum titer		small-scale
	GLC	*co substrate*
Mean	3.185	7.4
Variance	0.07605	0.08
Observations	2	2
Hypothesized Mean Difference	0	
df	2	
t Stat	-15.089703	
P(T<=t) one-tail	0.00218152	
t Critical one-tail	2.91998558	
P(T<=t) two-tail	0.00436304	
t Critical two-tail	4.30265273	

t-Test: Two-Sample Assuming Unequal Variances		
#2708 Maximum titer		small-scale
	GLC	*co substrate*
Mean	4.305	6.84
Variance	5E-05	0.0032
Observations	2	2
Hypothesized Mean Difference	0	
df	1	
t Stat	-62.88561	
P(T<=t) one-tail	0.0050413	
t Critical one-tail	6.31375151	
P(T<=t) two-tail	0.0101226	
t Critical two-tail	12.7062047	

t-Test: Two-Sample Assuming Unequal Variances		
#2705 Maximum titer		small-scale
	GLC	*co substrate*
Mean	6.25	8.62
Variance	0.0098	0.3528
Observations	2	2
Hypothesized Mean Difference	0	
df	1	
t Stat	-5.56608	
P(T<=t) one-tail	0.0565838	
t Critical one-tail	6.31375151	
P(T<=t) two-tail	0.1131676	
t Critical two-tail	12.7062047	

t-Test: Two-Sample Assuming Unequal Variances		
#2229 Yield		small-scale
	GLC	*co substrate*
Mean	0.064	0.148
Variance	3.2E-05	3.2E-05
Observations	2	2
Hypothesized Mean Difference	0	
df	2	
t Stat	-14.849242	
P(T<=t) one-tail	0.00225226	
t Critical one-tail	2.91998558	
P(T<=t) two-tail	0.00450453	
t Critical two-tail	4.30265273	

t-Test: Two-Sample Assuming Unequal Variances		
#2708 Yield		small-scale
	GLC	*co substrate*
Mean	0.0935	0.137
Variance	0.0001125	0.000002
Observations	2	2
Hypothesized Mean Difference	0	
df	1	
t Stat	-5.7491218	
P(T<=t) one-tail	0.05481825	
t Critical one-tail	6.31375151	
P(T<=t) two-tail	0.10963649	
t Critical two-tail	12.7062047	

t-Test: Two-Sample Assuming Unequal Variances		
#2705 Yield		small-scale
	GLC	*co substrate*
Mean	0.125	0.1535
Variance	0.000002	0.0001125
Observations	2	2
Hypothesized Mean Difference	0	
df	1	
t Stat	-3.766666	
P(T<=t) one-tail	0.08260158	
t Critical one-tail	6.31375151	
P(T<=t) two-tail	0.16520315	
t Critical two-tail	12.7062047	

t-Test: Two-Sample Assuming Unequal Variances		
#2229 Maximum titer		bioreactor
	GLC	*co substrate*
Mean	3.25	4.75
Variance	0.005	0.045
Observations	2	2
Hypothesized Mean Difference	0	
df	1	
t Stat	-9.486833	
P(T<=t) one-tail	0.03342936	
t Critical one-tail	6.31375151	
P(T<=t) two-tail	0.06685872	
t Critical two-tail	12.7062047	

t-Test: Two-Sample Assuming Unequal Variances		
#2708 Maximum titer		bioreactor
	GLC	*co substrate*
Mean	2.1	2.9
Variance	0	0.08
Observations	2	2
Hypothesized Mean Difference	0	
df	1	
t Stat	-4	
P(T<=t) one-tail	0.07797913	
t Critical one-tail	6.31375151	
P(T<=t) two-tail	0.15595826	
t Critical two-tail	12.7062047	

t-Test: Two-Sample Assuming Unequal Variances		
#2705 Maximum titer		bioreactor
	GLC	*co substrate*
Mean	1.65	2.9
Variance	0.005	0
Observations	2	2
Hypothesized Mean Difference	0	
df	1	
t Stat	-25	
P(T<=t) one-tail	0.01272561	
t Critical one-tail	6.31375151	
P(T<=t) two-tail	0.02545122	
t Critical two-tail	12.7062047	

t-Test: Two-Sample Assuming Unequal Variances		
#2229 Yield		bioreactor
	GLC	*co substrate*
Mean	0.07	0.085
Variance	0	0.00005
Observations	2	2
Hypothesized Mean Difference	0	
df	1	
t Stat	-3	
P(T<=t) one-tail	0.10241638	
t Critical one-tail	6.31375151	
P(T<=t) two-tail	0.20483276	
t Critical two-tail	12.7062047	

t-Test: Two-Sample Assuming Unequal Variances		
#2708 Yield		bioreactor
	GLC	*co substrate*
Mean	0.04	0.055
Variance	0	5E-05
Observations	2	2
Hypothesized Mean Difference	0	
df	1	
t Stat	-3	
P(T<=t) one-tail	0.10241638	
t Critical one-tail	6.31375151	
P(T<=t) two-tail	0.20483276	
t Critical two-tail	12.7062047	

t-Test: Two-Sample Assuming Unequal Variances		
#2705 Yield		bioreactor
	GLC	*co substrate*
Mean	0.033	0.052
Variance	0.000002	3.9506E-07
Observations	2	2
Hypothesized Mean Difference	0	
df	1	
t Stat	-17.362419	
P(T<=t) one-tail	0.01831304	
t Critical one-tail	6.31375151	
P(T<=t) two-tail	0.03662607	
t Critical two-tail	12.7062047	

Table S12 Welch t-test results obtained during small-scale production experiments. System Duetz® 24-deep well plates cultivation experiments were performed with 1.5 ml MTM medium and 50 g L^{-1} glucose. 6.25 g L^{-1} co-substrate were added during co-culture cultivations. Error bars indicate the deviation from the mean for n=2. Experiments performed with acetate are highlighted in yellow whereas formate cultivations are highlighted in grey.

t-Test: Two-Sample Assuming Unequal Variances			t-Test: Two-Sample Assuming Unequal Variances			t-Test: Two-Sample Assuming Unequal Variances			t-Test: Two-Sample Assuming Unequal Variances		
#2707 Maximum titer		small-scale	#2135 Maximum titer		small-scale	#2706 Maximum titer		small-scale	#2229 Maximum titer		small-scale
	GLC	*co substrate*		*GLC*	*co substrate*		*GLC*	*co substrate*		*GLC*	*co substrate*
Mean	2.145	2.8845	Mean	2.41	2.66	Mean	6.77	6.355	Mean	2.74	0.305
Variance	0.01805	4.05E-05	Variance	0.0002	0.1922	Variance	0.0338	0.59405	Variance	0.0338	5E-05
Observations	2	2	Observations	2	2	Observations	2	2	Observations	2	2
Hypothesized Mean Difference	0		Hypothesized Mean Difference	0		Hypothesized Mean Difference	0		Hypothesized Mean Difference	0	
df	1		df	1		df	1		df	1	
t Stat	-7.7754922		t Stat	-0.8060324		t Stat	0.74068772		t Stat	18.7169305	
P(T<=t) one-tail	0.04071409		P(T<=t) one-tail	0.2840559		P(T<=t) one-tail	0.29707281		P(T<=t) one-tail	0.01699037	
t Critical one-tail	6.31375151		t Critical one-tail	6.31375151		t Critical one-tail	6.31375151		t Critical one-tail	6.31375151	
P(T<=t) two-tail	0.08142819		P(T<=t) two-tail	0.5681118		P(T<=t) two-tail	0.59414562		P(T<=t) two-tail	0.03398074	
t Critical two-tail	12.7062047		t Critical two-tail	12.7062047		t Critical two-tail	12.7062047		t Critical two-tail	12.7062047	

t-Test: Two-Sample Assuming Unequal Variances			t-Test: Two-Sample Assuming Unequal Variances			t-Test: Two-Sample Assuming Unequal Variances			t-Test: Two-Sample Assuming Unequal Variances		
#2707 Yield		small-scale	#2135 Yield		small-scale	#2706 Yield		small-scale	#2229 Yield		small-scale
	GLC	*co substrate*		*GLC*	*co substrate*		*GLC*	*co substrate*		*GLC*	*co substrate*
Mean	0.043	0.057	Mean	0.0485	0.065	Mean	0.1305	0.113	Mean	0.0545	0.0045
Variance	8E-06	0.000002	Variance	5E-07	0.000072	Variance	0.0000125	0.0002	Variance	0.0000125	0.0000005
Observations	2	2	Observations	2	2	Observations	2	2	Observations	2	2
Hypothesized Mean Difference	0		Hypothesized Mean Difference	0		Hypothesized Mean Difference	0		Hypothesized Mean Difference	0	
df	1		df	1		df	1		df	1	
t Stat	-6.2609903		t Stat	-2.7405008		t Stat	1.69774938		t Stat	19.6116135	
P(T<=t) one-tail	0.05041437		P(T<=t) one-tail	0.11137147		P(T<=t) one-tail	0.16943737		P(T<=t) one-tail	0.01621664	
t Critical one-tail	6.31375151		t Critical one-tail	6.31375151		t Critical one-tail	6.31375151		t Critical one-tail	6.31375151	
P(T<=t) two-tail	0.10082873		P(T<=t) two-tail	0.22274294		P(T<=t) two-tail	0.33887474		P(T<=t) two-tail	0.03243328	
t Critical two-tail	12.7062047		t Critical two-tail	12.7062047		t Critical two-tail	12.7062047		t Critical two-tail	12.7062047	

t-Test: Two-Sample Assuming Unequal Variances			t-Test: Two-Sample Assuming Unequal Variances			t-Test: Two-Sample Assuming Unequal Variances			t-Test: Two-Sample Assuming Unequal Variances		
#2136 Maximum titer		small-scale	#1946 Maximum titer		small-scale	#2177 Maximum Titer		small-scale	#2196 Maximum Titer		small-scale
	GLC	*co substrate*		*GLC*	*co substrate*		*GLC*	*co substrate*		*GLC*	*co substrate*
Mean	2.32	2.27	Mean	0.075	0.011	Mean	2.69	0.46	Mean	0.605	0.0405
Variance	0.0032	0.005	Variance	0.00045	0.000002	Variance	0.0162	0.0018	Variance	0.00605	5E-07
Observations	2	2	Observations	2	2	Observations	2	2	Observations	2	2
Hypothesized Mean Difference	0		Hypothesized Mean Difference	0		Hypothesized Mean Difference	0		Hypothesized Mean Difference	0	
df	2		df	1		df	1		df	1	
t Stat	0.78086881		t Stat	4.25721667		t Stat	23.5062639		t Stat	10.2632123	
P(T<=t) one-tail	0.25831588		P(T<=t) one-tail	0.07343813		P(T<=t) one-tail	0.01353333		P(T<=t) one-tail	0.03091705	
t Critical one-tail	2.91998558		t Critical one-tail	6.31375151		t Critical one-tail	6.31375151		t Critical one-tail	6.31375151	
P(T<=t) two-tail	0.51663176		P(T<=t) two-tail	0.14687626		P(T<=t) two-tail	0.02706666		P(T<=t) two-tail	0.06183411	
t Critical two-tail	4.30265273		t Critical two-tail	12.7062047		t Critical two-tail	12.7062047		t Critical two-tail	12.7062047	

t-Test: Two-Sample Assuming Unequal Variances			t-Test: Two-Sample Assuming Unequal Variances			t-Test: Two-Sample Assuming Unequal Variances			t-Test: Two-Sample Assuming Unequal Variances		
#2136 Yield		small-scale	#21946 Yield		small-scale	#2177 Yield		small-scale	#2196 Yield		small-scale
	GLC	*co substrate*		*GLC*	*co substrate*		*GLC*	*co substrate*		*GLC*	*co substrate*
Mean	0.0465	0.045	Mean	0.0015	0	Mean	0.054	0.0085	Mean	0.012	0.001
Variance	5E-07	0.000002	Variance	0.0000005	0	Variance	8E-06	5E-07	Variance	0.000002	0
Observations	2	2	Observations	2	2	Observations	2	2	Observations	2	2
Hypothesized Mean Difference	0		Hypothesized Mean Difference	0		Hypothesized Mean Difference	0		Hypothesized Mean Difference	0	
df	1		df	1		df	1		df	1	
t Stat	1.34164079		t Stat	3		t Stat	22.0707419		t Stat	11	
P(T<=t) one-tail	0.20388458		P(T<=t) one-tail	0.10241638		P(T<=t) one-tail	0.0144124		P(T<=t) one-tail	0.02885794	
t Critical one-tail	6.31375151		t Critical one-tail	6.31375151		t Critical one-tail	6.31375151		t Critical one-tail	6.31375151	
P(T<=t) two-tail	0.40776917		P(T<=t) two-tail	0.20483276		P(T<=t) two-tail	0.0288248		P(T<=t) two-tail	0.05771588	
t Critical two-tail	12.7062047		t Critical two-tail	12.7062047		t Critical two-tail	12.7062047		t Critical two-tail	12.7062047	

Supplementary references

1. Djoumbou-Feunang, Y.; Pon, A.; Karu, N.; Zheng, J.; Li, C.; Arndt, D.; Gautam, M.; Allen, F.; Wishart, D. Significantly Improved ESI-MS/MS Prediction and Compound Identification. *Metabolites*. **2019** 9(4), 72.
2. Kellner, R.; Der Einfluss sexueller Reproduktion und Virulenz auf die Evolution und Speziation der biotrophen Brandpilzfamilie Ustilaginaceae. Dissertation. Germany: Ruhr-Universität Bochum. **2011.**
3. Leuchtle, B.; Microbial contamination of domestic heating oil : reasons and effects on fuel and tank. Dissertation. Germany: RWTH Aachen University. **2015**
